# *Plasmodium berghei* IMC1j interacts with δ-tubulin to orchestrate subpellicular microtubule organization in ookinetes

**DOI:** 10.1128/mbio.02669-25

**Published:** 2025-10-21

**Authors:** Gang He, Yonghui Feng, Dawei He, Yue Hu, Duo Wang, Sicong Wang, Xiaodan Sun, Shuangrui Shi, Tianyu Yang, Xueyuan Hu, Yaoguo Huang, Liwang Cui, Yaming Cao, Xiaotong Zhu

**Affiliations:** 1 Department of Immunology, College of Basic Medical Sciences, China Medical University 599277 , Shenyang, China; 2 National Clinical Research Center for Laboratory Medicine, Department of Laboratory Medicine, The First Hospital of China Medical University 159407, Shenyang, China; 3 Department of Pediatrics, The First Hospital of China Medical University 159407, Shenyang, Liaoning, China; 4 Department of Internal Medicine, Morsani College of Medicine, University of South Florida 824897, Tampa, Florida, USA; Rutgers-New Jersey Medical School, Newark, New Jersey, USA

**Keywords:** malaria, alveolins, inner membrane complex, δ-tubulin, fitness, invasion

## Abstract

Apicomplexan parasites possess specialized structures for cell invasion and replication, notably the inner membrane complex (IMC) and the subpellicular microtubules (SPMTs) situated beneath it. SPMTs are thought to interact with proteins embedded in or associated with the IMC. The cytosolic side of the IMC contains a network of proteins known as alveolins. This study focuses on a specific alveolar protein, *Plasmodium berghei* IMC1j (PbIMC1j). We found that PbIMC1j undergoes palmitoylation and is predominantly expressed in ookinetes. Gene knockout analysis revealed that the absence of PbIMC1j leads to defects in parasite asexual stage fitness and virulence, alters ookinete morphology, and impairs motility and/or infectivity in both ookinetes and sporozoites. Deletion analyses indicated that the C-terminal region containing the coiled-coil (CC) domain is required for proper targeting to and function within the IMC. Immunoprecipitation analysis of PbIMC1j demonstrated the role of the IMCp domain in mediating interactions with and stabilizing the expression of the IMC protein ISC1 and the SPMTs component δ-tubulin (TubD). In ookinetes devoid of TubD, the SPMTs were disorganized, mirroring the defects observed during the zygote-to-ookinete transition in the PbIMC1j-deficient strains. Overall, our study highlights the importance of the PbIMC1j protein in parasite fitness, SPMT organization, and ookinete motility mediated through its IMCp and CC domains.

**IMPORTANCE:**

Malaria parasites depend on an inner membrane complex (IMC) and subpellicular microtubules (SPMTs) to maintain their shape, motility, and ability to replicate. This study demonstrates that knocking out or disrupting the PbIMC1j protein adversely affects asexual stage fitness, virulence, and the morphology of ookinetes, while also decreasing the motility of ookinetes in *P. berghei*. Furthermore, PbIMC1j interacts and stabilizes the proteins ISC1 and δ-tubulin, underscoring its role in regulating the IMC and SPMTs.

## INTRODUCTION

Malaria is a potentially fatal infectious disease caused by *Plasmodium* species. In 2023, malaria led to approximately 597,000 deaths, with 80% of those being children under the age of 5 (1, 2). The malaria parasites have a complex life cycle that involves both a vertebrate host and a mosquito vector. When the mosquitoes take a blood meal, the gametocytes undergo gametogenesis, differentiating into gametes that fertilize to form zygotes. Within 24 hours, the spherical zygote elongates into a banana-shaped ookinete, gaining the ability to glide and migrate within the mosquito’s midgut. It then settles in the basal lumen, where thousands of sporozoites develop within an oocyst, preparing for transmission to another host (3–5). Antimalarial drugs targeting the parasite’s human-to-mosquito transmission could effectively reduce malaria’s prevalence in endemic populations when used in combination with one or two drugs active against the asexual blood stage. This approach is exemplified by clinical trials in which primaquine was combined with existing artemisinin-based combination therapies (6). A deeper understanding of the parasite’s unique biology is vital for identifying potential targets for these new treatments.

Apicomplexan biology is characterized by several features, including a distinctive mode of replication and invasion. The progeny of the parasite is assembled within the mother parasite, and invasion occurs through the formation of a tight-junction interface between the parasite and the host’s plasma membranes, allowing the parasite to enter the host cell (7, 8). Central to these processes is the inner membrane complex (IMC), a double-membrane organelle that plays a crucial role in the parasite’s life cycle, and a cortical cytoskeletal structure known as the subpellicular microtubules (SPMTs) (9). The IMC serves as an anchor for the parasite’s actin-myosin motor, which powers gliding motility and invasion (10, 11). Moreover, it acts as a scaffold for daughter cell formation during replication (12).

Supporting the IMC are SPMTs, which extend from the apical to basal end of the parasite, completing the cortical cytoskeleton. In organisms like *Plasmodium* and *Toxoplasma*, SPMTs are closely linked to the cytosolic side of IMC (13, 14). Research on protists with cortical microtubule-based cytoskeletons, such as *Euglenoids* and *Trypanosomes*, suggests that SPMTs likely interact with proteins embedded in or bound to the IMC (15–17). In *Plasmodium*, microtubules consist of α-, β-, γ-, and δ-tubulins. The α/β-tubulin dimers form protofilaments, while γ-tubulin aids in polymerization scaffolding. The function of δ- and ε-tubulin remains unknown, but δ-tubulin may contribute to the structure of the basal body (18, 19). Notably, there is significant amino acid conservation (83% and 87%) between human and *P. falciparum* α- and β-tubulins, while γ- and δ-tubulins show only about 33% homology (20), making them crucial targets for therapeutic agents.

Between SPMTs and the IMC, there exists a cortical cytoskeletal structure known as the subpellicular network (SPN) (21). This network includes a family of proteins referred to as alveolins (or the IMC1 family), which are homologous to the main components of the membrane skeleton found in algae and free-living protists. These proteins are thought to maintain cell shape and provide mechanical strength to the cell (22). In *Plasmodium* species, 13 conserved members of the IMC1 family have been identified, labeled as IMC1a through IMC1m (23). In *P. berghei*, research has demonstrated that disrupting the expression of individual IMC1 family members can lead to significant changes in the organism’s behaviors and physical characteristics. For instance, the loss of PbIMC1a, which is expressed during the sporozoite stage, PbIMC1b, which is expressed during the ookinete stage, and PbIMC1h, which is expressed during both the sporozoite and ookinete stages, results in reduced mechanical robustness, abnormal morphology, and impaired motility in both zoite stages (12, 24–26). Specifically, PbIMC1h has been shown to influence the gliding motility of both ookinete and sporozoite stages (27, 28). Additionally, IMC1g is required for the merozoite to sustain the mechanical stress of invasion in *P. falciparum* (29). Its conditional knockdown in *P. berghei* leads to deficiencies in late-stage schizogony, impaired gametogenesis, ookinete conversion deficiency, and significantly reduced ookinete gliding motility (30). In *Plasmodium falciparum*, the IMC1 family member PfIMC1l interacts with the actin-myosin motor, suggesting a direct role in regulating parasite motility (31). However, the role of alveolins in the interaction between the IMC and SPMTs remains unclear.

In this study, we demonstrate that PbIMC1j is a palmitoylated protein necessary for maintaining cell shape, gliding motility, and infectivity in both ookinetes and sporozoites. Domain deletion assays revealed that the C-terminal region harboring the coiled-coil (CC) domain is required for protein localization and function. Furthermore, we establish that PbIMC1j interacts with and stabilizes the microtubule component δ-tubulin (TubD) through its IMCp domain, thereby contributing to SPMT assembly. Notably, TubD knockout recapitulates the stage-specific phenotypic defects of PbIMC1j in sexual stages. Collectively, our findings establish PbIMC1j as an important regulator across multiple stages of the *P. berghei* life cycle.

## RESULTS

### PbIMC1j is a conserved palmitoylated IMC1 protein enriched in ookinetes and targeted to the IMC/SPN through lipid modification

The *P. berghei* IMC1j (PbIMC1j) is a protein consisting of 623 amino acids. It features a conserved IMCp domain (Pfam Accession No. pfam12314; E-value: 3.9e-25; amino acids [aa] 134–228) and a C-terminal coiled-coil (CC) segment (aa 403–434). Phylogenetic analysis revealed high sequence conservation among apicomplexan parasites, including *Toxoplasma*, *Cystoisospora*, and *Eimeria* species (Fig. S1A). Within the *Plasmodium* genus, PbIMC1j shares 50%–82% sequence identity with orthologs from other species (Fig. S1B and C), suggesting functional conservation.

**Fig 1:**
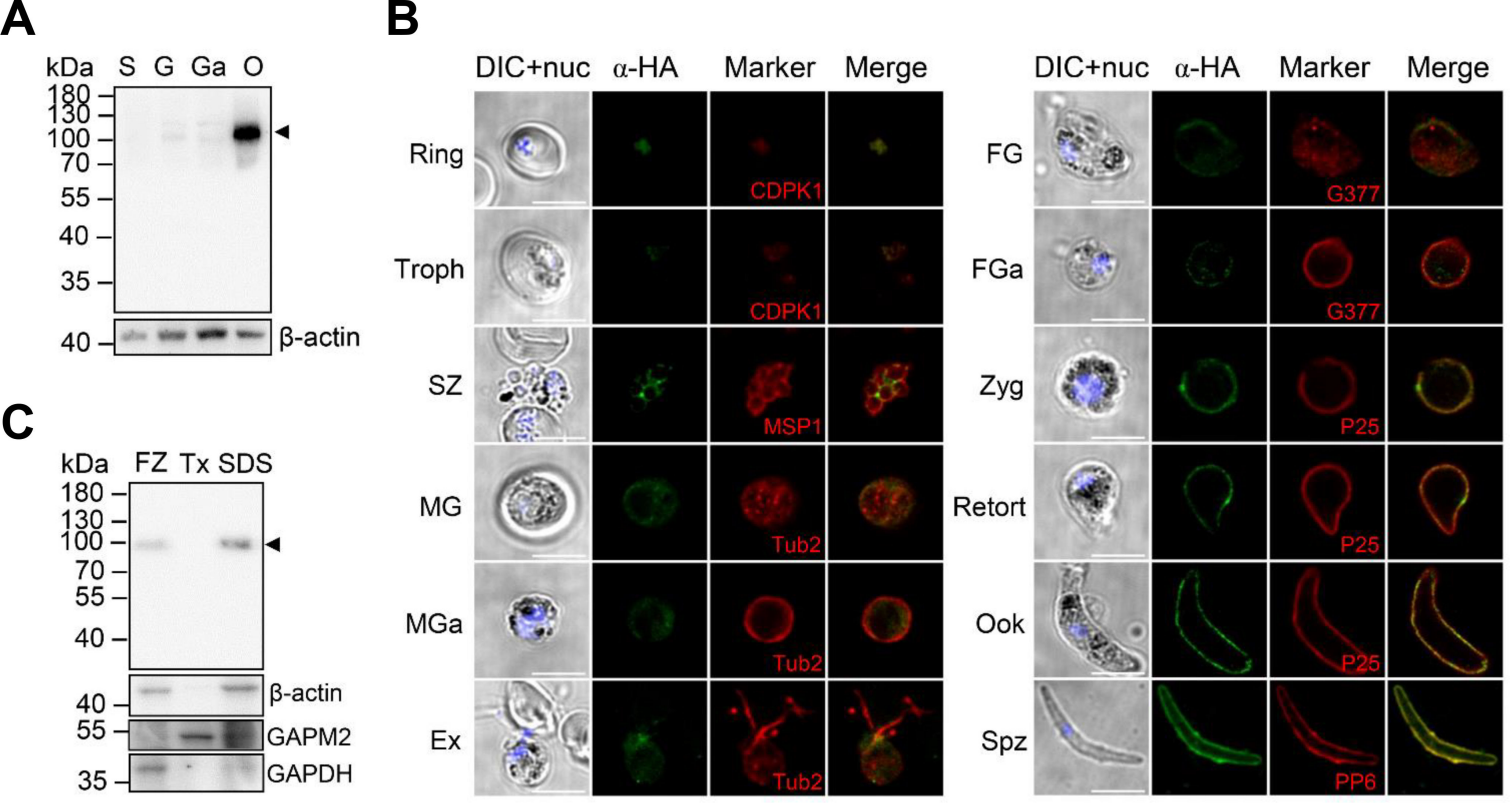
PbIMC1j is a membrane-bound IMC protein enriched in ookinetes. (**A**) Immunoblotting analysis shows the PbIMC1j-HA protein in different stages: schizont (S), gametocyte (G), activated gametocyte (Ga), and ookinete (O). The PbIMC1j-HA fusion protein (arrowhead) was detected using an anti-HA antibody. β-actin serves as the loading control. (**B**) Immunofluorescence analysis (IFA) of PbIMC1j-HA protein in the life cycle of PbIMC1j^HA-^*^glmS^* parasites. The observed stages include trophozoite (Thoph), schizont (SZ), male and female gametocyte (MG and FG), activated male and female gametocyte (MGa and FGa), exflagellated male gametocyte (Ex), zygote (Zyg), ookinete (Ook), and sporozoite (Spz). Images include differential interference contrast (DIC), DAPI-stained nuclei (blue), PbIMC1j-HA (green), and colocalization markers (red). Scale bars: 5 µm. All images were acquired using identical microscopy settings. (**C**) Solubility profile of the PbIMC1j-HA protein in ookinetes under sequential extraction conditions: freeze-thaw (FZ), 1% Triton X-100 detergent (Tx), and 2% SDS detergent (SDS). The PbIMC1j-HA protein is indicated by a black arrowhead, with β-actin as a loading control. Data in (**A–C**) are representative of three independent biological replicates.

To examine the expression and localization of PbIMC1j, we generated a transgenic parasite line (PbIMC1j^HA-^*^glmS^*) using CRISPR–Cas9 to tag the endogenous *pbimc1j* locus with a C-terminal HA epitope and a *glmS* ribozyme sequence (Fig. S2A). Diagnostic PCR confirmed correct integration (Fig. S2B). Western blot analysis using an anti-HA monoclonal antibody (mAb) detected a band of approximately 100 kDa, larger than the predicted molecular mass of 76.9 kDa (Fig. S2C). This discrepancy may be attributed to potential N-glycosylation at residues N284, N306, N338, N378, N518, N580, and N611 within the CPDc domain (NetNGlyc prediction scores: 0.50–0.63), which can reduce electrophoretic mobility. PbIMC1j-HA expression was highest in ookinetes (Fig. 1A). Indirect immunofluorescence assays (IFAs) showed that PbIMC1j was detectable in the parasite cytosol from the ring stage onward and exhibited cortical localization from schizonts to ookinetes, consistent with typical IMC/SPN patterning (Fig. 1B). Solubility assays further revealed that PbIMC1j was predominantly present in the SDS-soluble fraction in ookinetes (Fig. 1C), a property that is consistent with that of many membrane-associated proteins, including components of the IMC/SPN.

**Fig 2:**
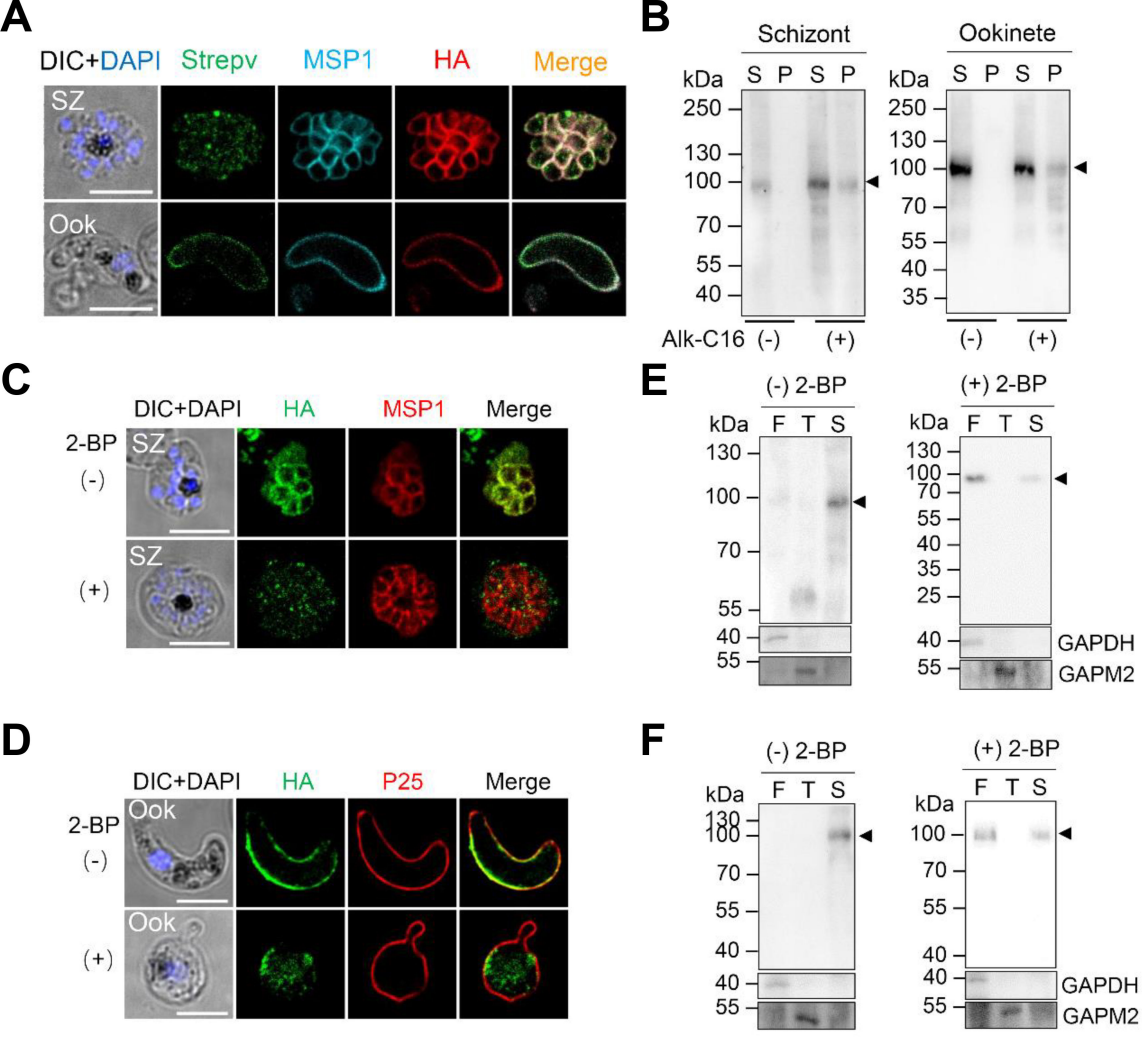
Click chemistry assay of PbIMC1j protein in schizonts and ookinetes. (**A**) The localization of the palmitoylated PbIMC1j protein in the schizont (SZ) and ookinete (Ook) stages was identified using click chemistry. Parasites were metabolically labeled with alkynyl palmitic acid (Alk-C16) and subsequently stained with Alexa Fluor 488-conjugated streptavidin (Strepv, green), anti-MSP1 serum or anti-P25 serum (cyan), and anti-HA mAb (red). Scale bars: 5 µm. (**B**) Western blot analysis of palmitoylated PbIMC1j proteins in schizonts (left panel) and ookinetes (right panel). The Alk-C16 labeled (+) proteins were detected using anti-HA mAb. S, immunoprecipitation (IP) supernatant; P, IP elution. The arrowhead indicates the PbIMC1j-HA protein. (**C**) IFA of PbIMC1j^HA-^*^glmS^* parasites at the schizont stage (SZ) after treatment without (−) or with 100 µM of 2 BP (+). Scale bars: 5 µm. (**D**) PbIMC1j-HA localization in ookinetes treated without (−) or with (+) 100 µM of 2 BP. Scale bars: 5 µm. (**E**) Solubility of the PbIMC1j-HA in schizonts following 2 BP treatment. Sequential extraction was performed with freeze-thaw (**F**), 1% Triton X-100 (T), and 2% SDS (S). GAPDH (soluble) and GAPM2 (membrane) serve as fractionation controls. (**F**) Solubility profile of PbIMC1j-HA protein in ookinetes after 2 BP treatment. Extraction conditions as in (**D**). All panels are representative of three biological replicates.

PbIMC1j contains six predicted palmitoylation sites at cysteines 6, 13, 14, 146, 257, and 370 (Table S1). To test if PbIMC1j is palmitoylated, we metabolically labeled schizont and ookinete lysates from PbIMC1j^HA-^*^glmS^* parasites with alkynyl palmitic acid (Alk-C16) (32). Click chemistry assays confirmed that PbIMC1j is palmitoylated at both stages, as evidenced by a distinct band in the pellet fraction of labeled samples that is absent in the no-click controls (Fig. 2A and B). Treatment with the palmitoylation inhibitor 2-bromopalmitate (2 BP) resulted in diffuse cytosolic staining of PbIMC1j-HA, in contrast to the cortical localization in mock-treated parasites (Fig. 2C). Consistent with previous reports (33), 2 BP treatment also nearly completely inhibited ookinete development (Fig. 2D). The mislocalization of PbIMC1j following inhibition suggests that palmitoylation is essential for its correct targeting to the IMC/SPN. To further investigate this, we performed detergent fractionation assays. In untreated parasites, PbIMC1j was predominantly SDS-soluble. However, in 2 BP-treated parasites, the protein was redistributed and primarily found in the freeze-/thaw-soluble fraction (Fig. 2E and F). This shift in solubility indicates a loss of membrane association, consistent with impaired palmitoylation. Collectively, these data demonstrate that PbIMC1j is palmitoylated and that this post-translational modification is necessary for its membrane association and targeting to the IMC/SPN.

### PbIMC1j is required for *P. berghei* virulence and asexual stage fitness

Although previous studies reported that PbIMC1j is dispensable for asexual blood-stage development (34, 35), we further investigated its functional domains. Using CRISPR–Cas9, we generated knockout (KO) mutants lacking the entire coding region (bp 22–1869) and C-terminal deletion (Del) mutants (aa 814–1869) of *pbimc1j* in the *P. berghei* ANKA wild-type (WT) strain (Fig. S3A). Successful mutagenesis was confirmed by PCR and Western blot (Fig. S3B through F). IFA showed complete loss of HA signal in KO parasites, while Del mutants exhibited diffuse cytosolic staining in schizonts but retained peripheral localization in ookinetes (Fig. S3G), indicating that the C-terminus is essential for correct SPN targeting in schizonts.

We next assessed virulence using two mouse models: C57BL/6 J mice, which are susceptible to experimental cerebral malaria (ECM), and BALB/c mice, which develop severe anemia and hyperparasitemia. In C57BL/6 J mice, parasitemia was significantly lower in PbIMC1j-Del–infected mice than in WT-infected mice from day 5 post-infection (p.i.) onward (Fig. S3H). Notably, 80% of WT-infected mice succumbed to ECM by days 8–10 p.i., whereas all PbIMC1j-Del–infected mice survived beyond day 14 p.i. (Fig. S3I). This attenuation occurred despite comparable splenic activation of IFN-γ^+^ Th1 and granzyme B^+^ CD8^+^ T cells in both groups at day 5 p.i. (Fig. S4A and B) and similar levels of brain-infiltrating CD8^+^ T cells (Fig. S4E and F). In BALB/c mice, WT infection caused 100% mortality by day 24 p.i., whereas 70% of Del-infected mice survived (Fig. S3J and K). Together, these data demonstrate that PbIMC1j-Del parasites are attenuated in virulence, independent of initial T cell activation, thereby establishing that the C-terminal domain is important for blood-stage fitness and pathogenicity.

### PbIMC1j enables ookinete conversion and cytoskeletal integrity through its C-terminal domain

**Fig 3:**
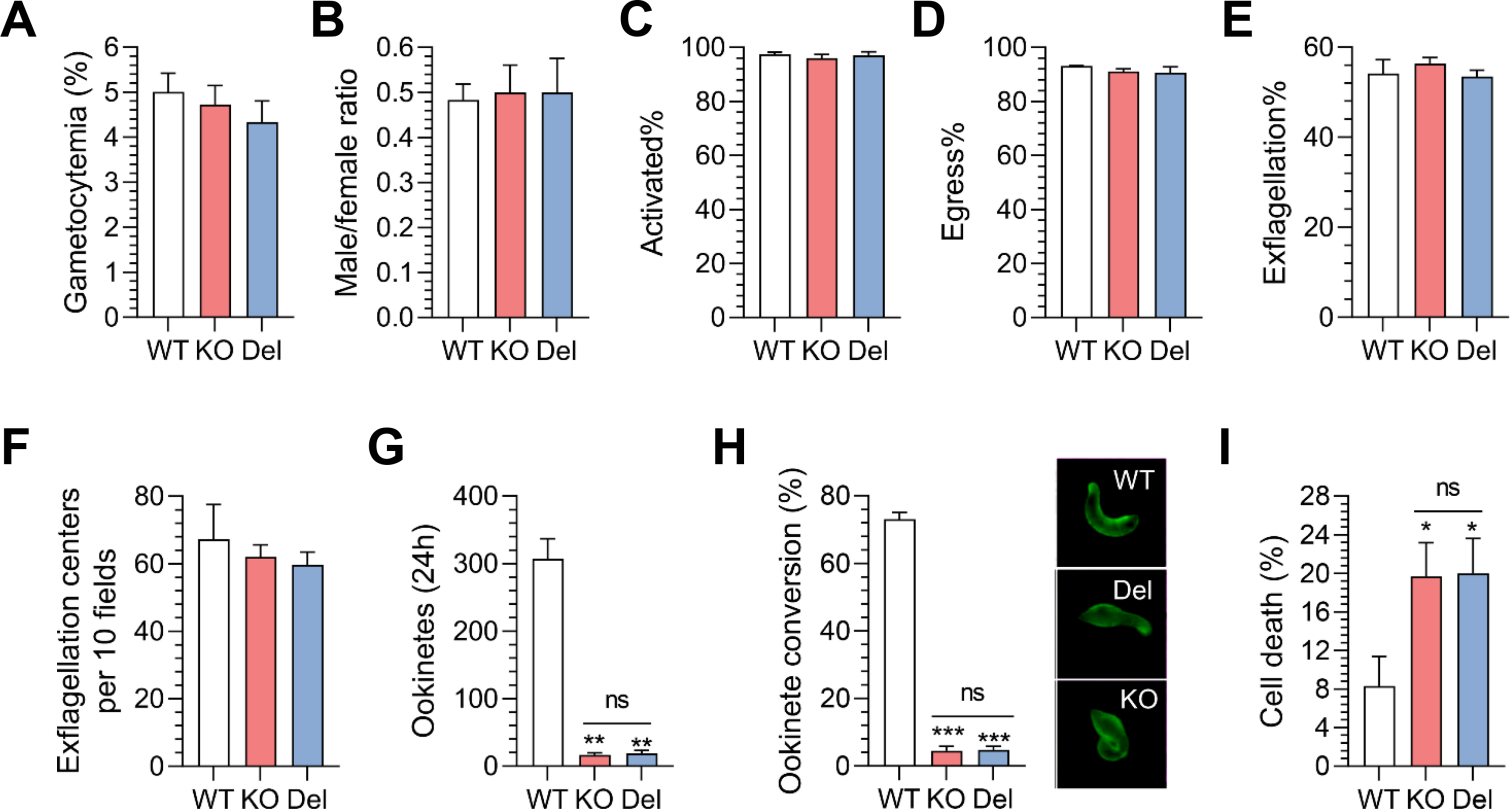
The C-terminal region of PbIMC1j is required for regulating ookinete morphology and tensile strength. (**A**) Gametocytemia levels in the WT, KO, and Del parasite strains were measured 3 days post-infection (dpi). (**B**) The male/female ratios indicate the comparative gender distributions among the aforementioned parasite strains at 3 dpi. (**C**) Quantifies the percentage of activated microgametocytes for each strain at 3 dpi. (**D**) The proportions of microgametocytes forming gametes (%) in WT, KO, and Del parasites are presented at 3 dpi. (**E**) The percentages of exflagellated parasites post-activation and (**F**) compare the number of exflagellation centers among the WT, KO, and Del groups, where the mean number of centers (± SD) is derived from two independent experiments, validated by Student’s *t*-test with significant differences marked (*, *P* < 0.05). (**G**) The matured ookinete count for WT, KO, and Del parasites. (**H**) Ookinete conversion rates (%) based on Pbs21-positive parasites, with representative IFA images in the right panel. (**I**) Cell viability post-hypo-osmotic shock treatment reveals cell death inversely correlates with tensile strength. Data are presented as mean ± SD from two independent experiments (*n* = 100). Significant differences for KO/Del compared to WT (*, *P* < 0.05), while KO vs Del remained not significant (ns). All data are representative of three biological replicates.

While gametocyte development was unaffected, both KO and Del mutants exhibited severe defects in ookinete formation (Fig. 3A through H). Only 6.2% of KO and 5.4% of Del parasites formed mature ookinetes, representing >93% reduction compared to WT (Fig. 3G). Following 24 hours of *in vitro* culture, 73.1% of WT parasites developed characteristic banana-shaped ookinetes, whereas only 4.8% of KO and 4.8% of Del parasites attained mature morphology (Fig. 3H). Most mutants were arrested at zygote or bulb-shaped stages (Fig. 3H). Conditional knockdown via glucosamine treatment (2.5 mM) in the PbIMC1j^HA-^*^glmS^* line, a system previously adapted for murine models (36), led to a 17.2% reduction in ookinete conversion efficiency, without associated morphological abnormalities (Fig. S5), implying that the C-terminus specifically regulates morphogenetic maturation. Moreover, hypo-osmotic stress assays (37) revealed an 11% increase in cell death in KO and Del ookinetes (Fig. 3I). The increased sensitivity to hypo-osmotic stress is consistent with a defect in the structural integrity and mechanical stability of the mutant ookinetes.

**Fig 4:**
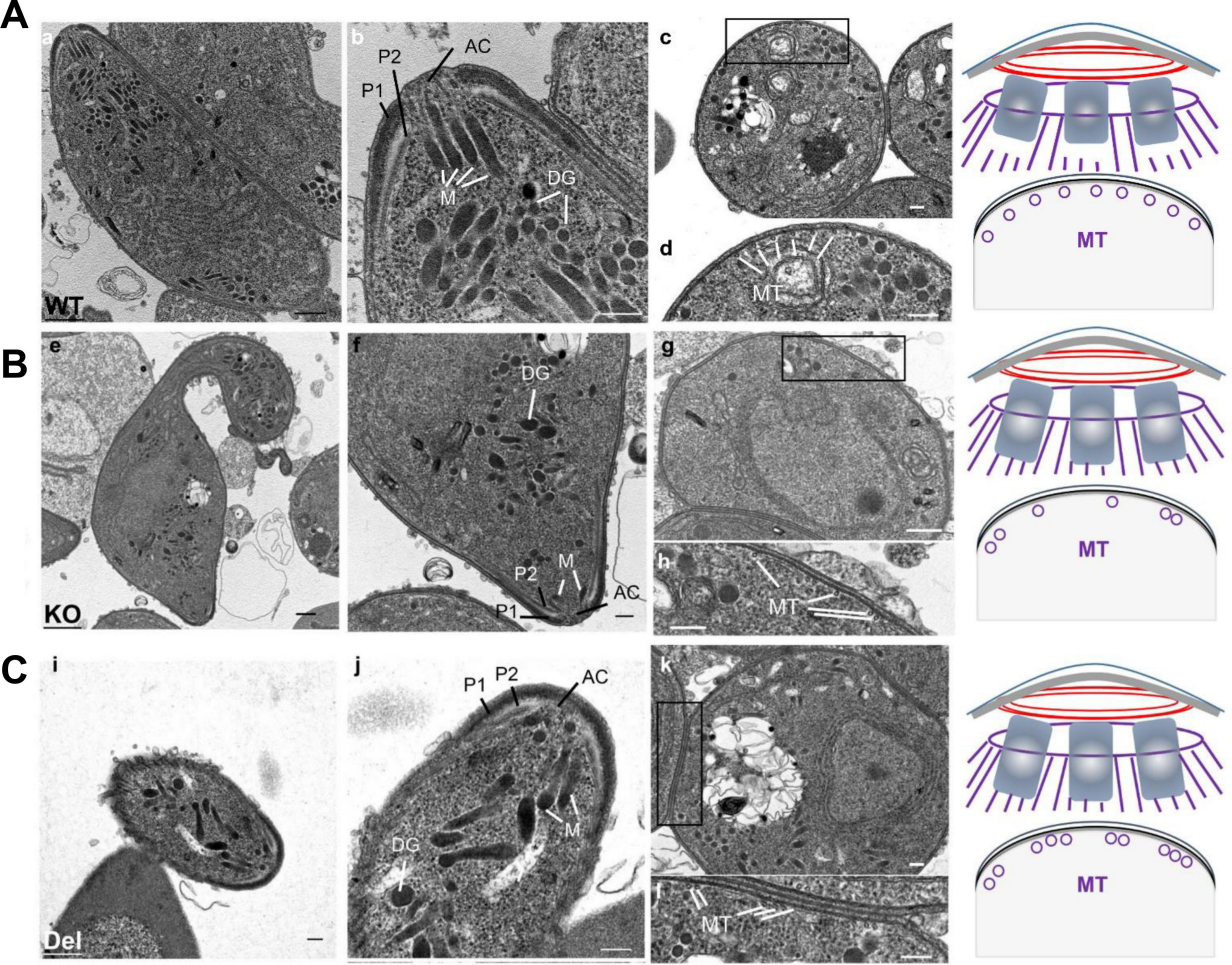
Ultrastructural analysis of WT (**A**), PbIMC1j KO (**B**), and Del parasites (**C**) at the ookinete stage is presented. Longitudinal sections of the WT (**a and b**), KO (**e and f**), and Del (**i and j**) ookinetes exhibit key structural features, including the apical complex (AC), micronemes (M), dense granules (DG), and polar rings (P1 and P2). Cross-sectional images of the anterior complex in the WT (**c and d**), KO (**g and h**), and Del (**k and i**) reveal comparable substructures encompassing the parasite plasma membrane (PPM) and the inner membrane complex (IMC). Additionally, subpellicular microtubules (MT) are explicitly indicated. The scale bars measure 500 nm (black) and 200 nm (white). Accompanying schematics in the right panel illustrate the configuration of the apical complex and the spatial arrangement of subpellicular microtubules in WT, KO, and Del parasites. Data are representative of two independent experiments (WT, *n* = 34; KO, *n* = 28; Del, *n* = 33 ookinetes analyzed).

Transmission electron microscopy (TEM) analysis of 12 hour ookinetes demonstrated conserved apical complex organization across all genotypes, comprising (i) the IMC-derived apical polar ring and (ii) secondary polar rings functioning as nucleation sites for SPMT assembly (Fig. 4A through C; Fig. S6A). In contrast to the ordered microtubule bundles observed in WT parasites (Fig. 4A, c and d; Fig. S6A, b and c), KO and Del mutants exhibited severely disrupted SPMT arrays (Fig. 4B, g and h; 4C, i through k; Fig. S6A, e and f; S6A, h and i). This defect was fully penetrant: all 61 high-quality TEM sections examined from mutant parasites (*n* = 28 KO; *n* = 33 Del) showed clear microtubule disorganization (Fig. 4; Fig. S6). Quantitative analysis further confirmed a significant increase in the distance between adjacent microtubule pairs in mutants compared to WT (Fig. S6B). Collectively, these findings establish that PbIMC1j directs SPMT assembly and confers mechanical stability for ookinete development through its C-terminal domain.

### PbIMC1j mediates gliding motility and mosquito transmission efficiency

**Fig 5:**
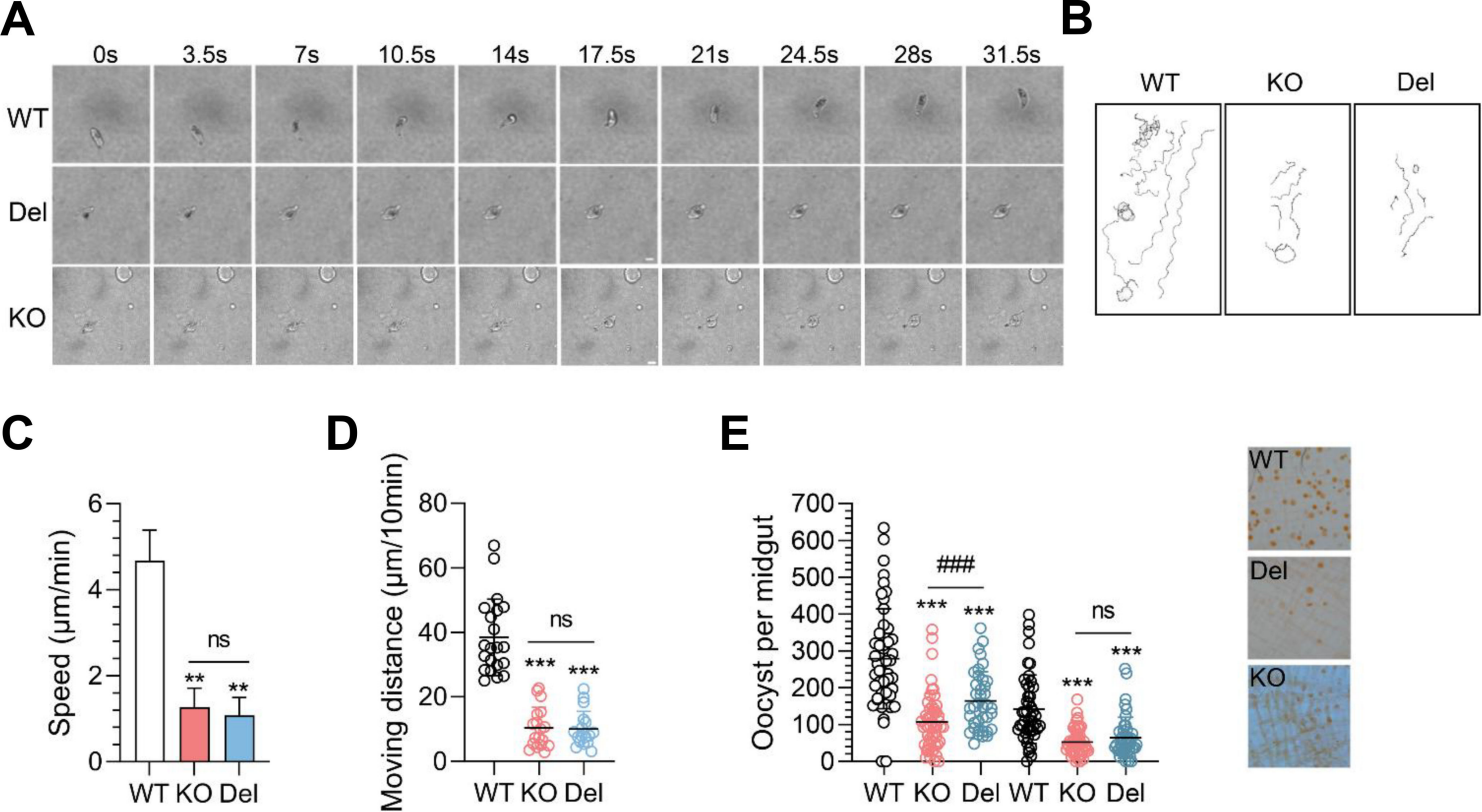
Analysis of ookinete motility and infectivity in PbIMC1j transgenic parasites. (**A**) Representative time-lapse images of gliding ookinetes from WT, KO, and Del parasites. Scale bar: 5 µm. (**B**) Trajectories of individual parasites over 30 minute period. (**C**) Gliding speed was measured in μm/sec, showing significant differences between KO/Del and WT (**, *P* < 0.01), but not between KO and Del (ns, not significant). (**D**) Moving distances calculated *via* ImageJ indicated significant differences for KO/Del compared to WT (***, *P* < 0.001), while KO vs Del remained not significant (ns). WT, *n* = 21; Del, *n* = 17; KO, *n* = 18. Data in (**A–D**) are representative of three independent experiments. (**E**) The number of oocysts in the midguts of mosquitoes infected with WT, KO, and Del strains is shown at 10 days post-infection (dpi). Each dot represents the count of oocysts in an individual mosquito, and horizontal bars indicate the average number and standard deviation. Statistical assays between KO or Del to WT: ***, *P* < 0.001; Statistical assays between KO and Del: ns, not significant; ###, *P* < 0.001. The data reflect two independent feeding experiments: Experiment 1 included WT (*n* = 51), Del (*n* = 38), and KO (*n* = 51); Experiment 2 included WT (*n* = 47), Del (*n* = 44), and KO (*n* = 50).

Since ookinetes are motile and invasive stages, we conducted further examinations on the gliding motility of WT, KO, and Del ookinetes using a time-lapse assay. The WT ookinetes displayed normal corkscrew-like movement, with an average speed of approximately 4.6 µm/min. In contrast, the KO and Del ookinetes moved significantly slower, with average speeds of 1.3 µm/min and 1.1 µm/min, respectively (Fig. 5A through C; Movies S1 to S3). Notably, the movement distances of the KO and Del ookinetes were significantly shorter than those of WT ookinetes, indicating that their gliding movement was less effective (Fig. 5D; Movies S1 to S3).

To evaluate the infectivity of KO and Del ookinetes, a direct mosquito feeding assay was performed using mice infected with WT, KO, and Del parasites. Ten days post-infection (dpi), the prevalence of infection and the density of oocysts in the mosquito midgut were evaluated. There were no statistically significant differences in infection prevalence between the mutants and WT parasites: KO was 96.1%, Del was 96.6%, and WT was 97% (Table 1). Additionally, the KO and Del mosquitoes exhibited a 62.0% and 45.6% reduction in oocyst density, respectively, indicating ookinetes had a reduced ability to invade or undergo the early stages of oocyst development (Fig. 5E and Table 1).

**TABLE 1:** Transmission of PbIMC1j KO and Del parasites in *Anopheles stephensi*

	WT		KO		Del	
	Exp1	Exp2	Exp1	Exp2	Exp1	Exp2
Mosquito-infected/dissected	49/51	46/47	49/51	48/50	38/38	41/44
Prevalence of infection (%)^*a*^	96.1	97.9	96.1	96	100	93.2
Mean prevalence (%)		97		96.1		96.6
Reduction in prevalence (%)^*b*^				0.9^ns^		0.4^ns^
Oocyst intensity^*c*^	278.6	141.5	106.9	52.6	164.1	64.5
SEM^*d*^	19.0	13.5	10.9	5.0	13.0	8.3
Mean oocyst intensity		210.1		79.8		114.3
Reduction in oocyst intensity (%)^*e*^				62.0^***^		45.6^***^
Sporozoite/SG^*f*^	10,851	7031	136	98	3,485	4,237
Mice positive after 3,000 sporozoite injection	3/3	3/3	0/5	0/5	2/3	2/3
Day of 0.5–2% parasitemia after sporozoite injection	7.3	6.3	-	-	18.5	11.5

^*a*^ Infection prevalence: calculated by dividing the number of mosquitoes with oocysts by the total number of mosquitoes dissected in each group and then multiplying by 100%.

^*b*^ The percent reduction in prevalence was calculated using the formula: (% mean prevalence of WT − % mean prevalence of KO/Del). Fisher’s exact test was applied; “ns” indicates not significant.

^*c*^ Mean oocysts per mosquito: refers to the average number of oocysts found in the midgut of mosquitoes.

^*d*^ The standard error of the mean.

^*e*^ Percent reduction in oocyst intensity: calculated as: [(mean oocyst intensity of WT − mean oocyst intensity of KO/Del)/mean oocyst intensity of WT] × 100%. Mann-Whitney *U* test was used; *** *P* < 0.001. Exp, experiment.

^*f*^ Mean number of 10 salivary glands.

The sporozoite load in the salivary glands was significantly lower in mosquitoes that fed on mice infected with the KO and Del strain than those that fed on WT mice (Table 1). To evaluate the viability of the sporozoites, we intravenously injected purified sporozoites from the salivary glands of both the WT, KO, and Del groups into naïve BALB/c mice, using a dosage of 3,000 sporozoites per mouse (isolated at 19 dpi). We found that all mice (6/6) inoculated with WT sporozoites developed blood-stage infections, whereas only 66.7% (4/6) of mice inoculated with Del sporozoites became infected, and none (0/10) of those receiving KO sporozoites developed parasitemia (Table 1). Moreover, the prepatent period was significantly longer in Del-infected mice (11.5–18.5 dpi) than in WT-infected controls (6.3–7.3 dpi) (Table 1), indicating that PbIMC1j-deficient sporozoites are either less viable or compromised in their abilities, potentially due to impaired liver-stage invasion, development, or merozoite egress from hepatocytes, which hinders their ability to establish a productive infection. Together, these results demonstrate that PbIMC1j is essential for productive gliding motility, mosquito midgut invasion, and sporozoite infectivity.

### PbIMC1j interacts with δ-tubulin with its IMCp domain

**Fig 6:**
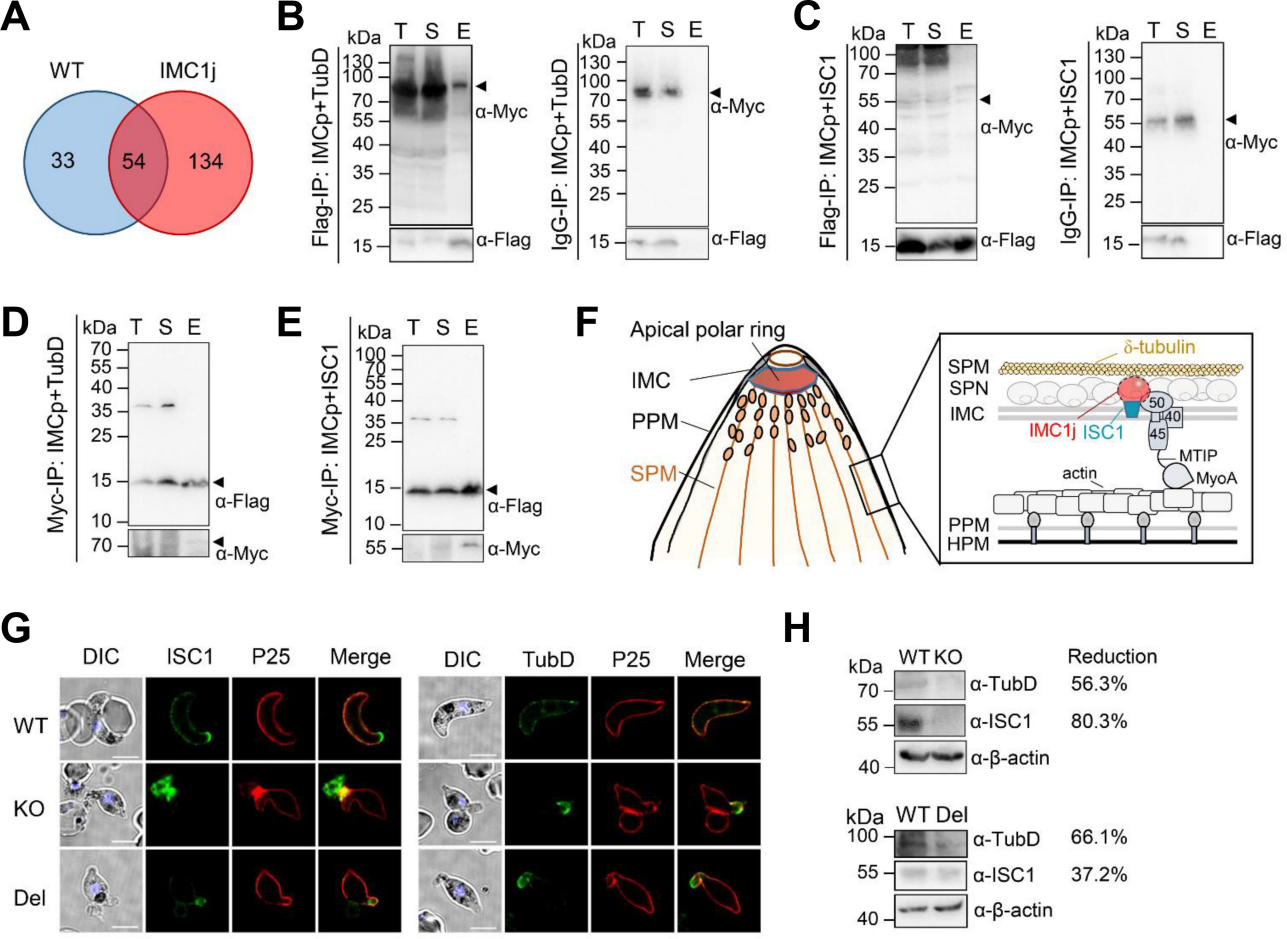
Interaction of PbIMC1j with ISC1 and TubD during ookinete stages. (**A**) A Venn diagram shows protein precipitation from PbIMC1j-HA immunoprecipitation (IP) analyzed by LC-MS/MS in parental *P. berghei* parasites (WT) and PbIMC1j^HA-^*^glmS^* mutants (IMC1j). (**B, C**) Pull-down assays with Flag-conjugated or IgG-conjugated beads explored interactions between Flag-IMCp and either TubD-Myc-His (**B**) or ISC1-Myc-His (**C**). Immunoblots were probed using anti-Myc and anti-Flag mAb. T, total lysate; S, supernatant; E, elution. Flag-IMCp, 13.8 kDa; TubD-Myc-his, 85.7 kDa; ISC1-Myc-his, 60.2 kDa. (**D, E**) Reciprocal co-IP using anti-Myc beads with TubD-Myc-His (**D**) or ISC1-Myc-His (**E**). Blots were probed with anti-Flag (top) and anti-Myc (bottom), using the same notation for T, S, and E. (**F**) Model of the PbIMC1j domain architecture and its interactions with TubD and ISC1 within the IMC, illustrating associations with the parasite plasma membrane (PPM), subpellicular microtubules (SPMTs), and host plasma membrane (HPM). (**G**) Immunofluorescence on WT, KO, and Del ookinetes stained with anti-P25 sera (red) and either anti-ISC1 (green, left) or anti-TubD (green, right) is presented, with a scale bar of 5 µm. (**H**) Immunoblotting assessed ISC1 (56.5 kDa) and TubD expression (82.1 kDa) in PbIMC1j KO and Del strains, using β-actin as a loading control. Data in (**B–E**) and (**H**) are representative of three independent experiments; (**G**) is representative of two experiments.

To identify the binding partners of PbIMC1j, we conducted a co-immunoprecipitation (co-IP) experiment using lysates from PbIMC1j^HA-^*^glmS^* ookinetes with HA-conjugated beads. Probing with anti-HA antibodies confirmed that we successfully purified the bait PbIMC1j-HA protein (Fig. S7A). Subsequent LC-MS/MS analysis identified 134 unique proteins specifically precipitated with the HA-beads compared to the control beads (Fig. 6A; Table S2). Based on the localization of PbIMC1j to the IMC/SPN and its role in SPMT organization, we prioritized alpha-tubulin-1 (TubA), δ-tubulin (TubD), and inner membrane suture protein 1 (ISC1) as candidate interactors (Table S2). However, we did not detect a direct interaction between TubA and PbIMC1j (Fig. S7B).

To validate interactions with TubD and ISC1 and determine whether the IMCp domain mediates these associations, we co-expressed a Flag-tagged IMCp domain of PbIMC1j with either Myc-His-tagged ISC1 or TubD in HEK293T cells and performed co-IP using anti-Flag beads (Fig. S6C). Flag-IMCp co-precipitated protein bands comigrating with TubD and ISC1, which were absent in control precipitations (Fig. 6B and C; Fig. S7D and E). Reciprocally, anti-Myc antibody beads coprecipitated Flag-IMC1p protein (Fig. 6D and E; Fig. S7F). These results indicate that PbIMC1j can interact with the ISC1 and TubD with its IMCp domain in *P. berghei* parasites (Fig. 6F).

We further investigated the localization and expression levels of these two IMC-tethered proteins in PbIMC1j-KO and -Del mutants using anti-ISC1 and anti-TubD sera, respectively (Fig. S7G and H). Our results showed that the signals for ISC1 and TubD were significantly reduced in the IMC/SPN of both KO and Del parasites (Fig. 6G). Additionally, we found that the expression levels of ISC1 and TubD proteins were diminished in KO and Del parasites, with reductions ranging from 37.2% to 80.3% (Fig. 6H; Fig. S7I and J). These findings indicate that PbIMC1j is essential for properly targeting and stable expression of ISC1 and TubD proteins in the IMC/SPN.

### TubD deletion phenocopies PbIMC1j loss-of-function phenotypes in SPMT organization and ookinete maturation

**Fig 7:**
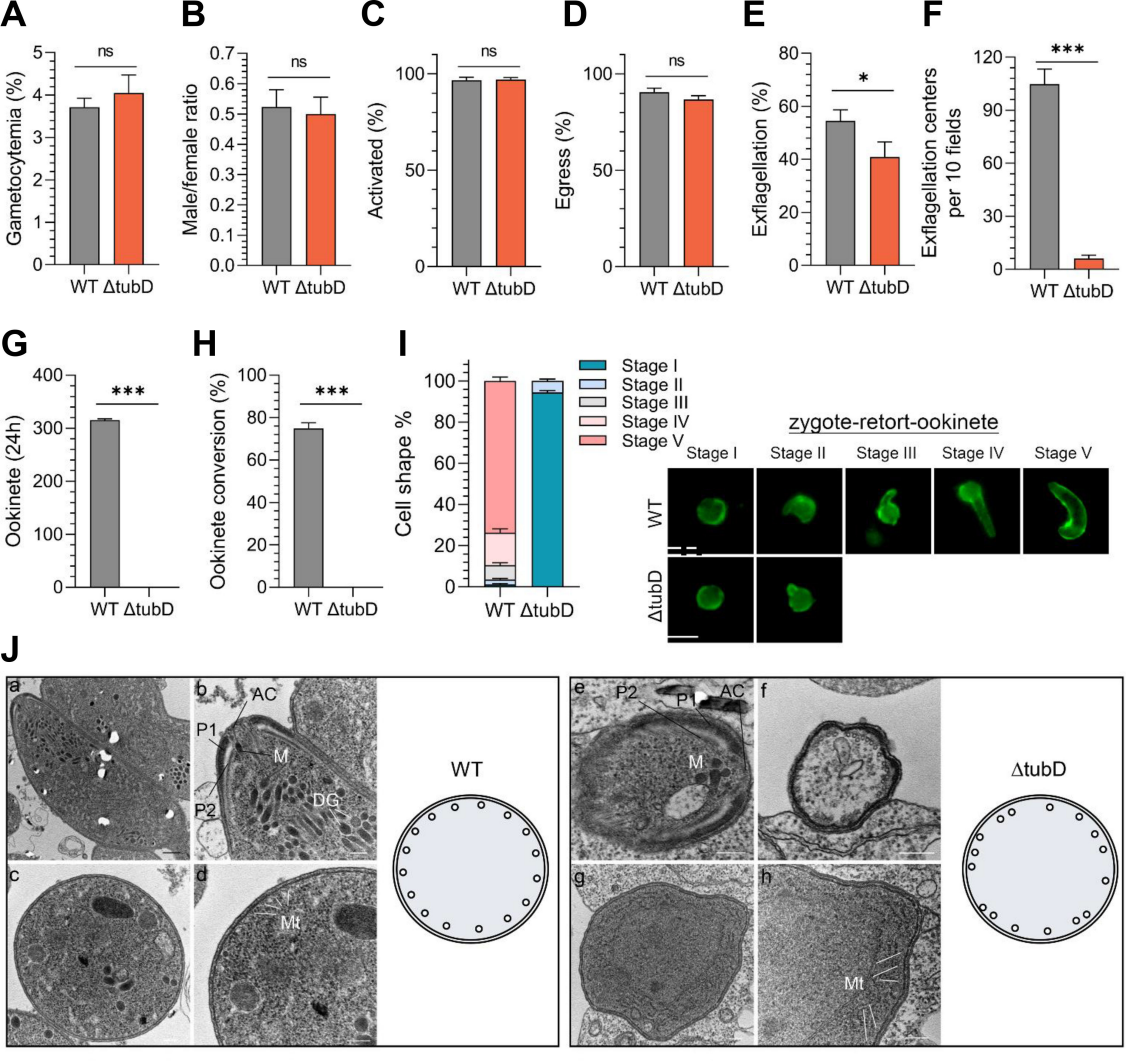
TubD is essential for male gamete formation, SPMT organization, and ookinete development. (**A–I**) Phenotypic characterization of sexual stages in ΔtubD parasites. Quantification of (**A**) gametocytemia; (**B**) male-to-female ratio; (**C**) gametocyte activation; (**D**) gamete formation; (**E**) exflagellation percentage; (**F**) number of exflagellation centers; (**G**) mature ookinete counts; (**H**) ookinete conversion rates (based on Pbs21-positive parasites); (**I**) morphological stages during zygote-to-ookinete differentiation in WT and ΔtubD strains. Data represent mean ± SD; *n* = 3 biological replicates; Student’s *t*-test; *, *P* < 0.05; ***, *P* < 0.001; ns, not significant. Representative IFA images shown at right. (**J**) Transmission electron micrographs of WT (left) and ΔtubD (right) ookinetes. Longitudinal sections (**a–b, e**) show apical complex (AC), micronemes (M), dense granules (DG), and polar rings (P1, P2). Cross-sections (**c–d, f–h**) reveal disorganized subpellicular microtubules (MT) in ΔtubD parasites. Scale bars: 500 nm (black); 200 nm (white). Schematics summarize microtubule arrangement. All data are representative of three independent experiments.

To investigate whether TubD contributes to the cytoskeletal defects observed in PbIMC1j-deficient parasites, we generated a *tubD* knockout (ΔtubD) strain in *P. berghei* using a homology-directed repair strategy (Fig. S8A). Successful gene deletion was confirmed by diagnostic PCR and Western blot analysis with anti-TubD sera (Fig. S8B and C). Consistent with prior reports (34, 35), ΔtubD parasites exhibited normal blood-stage replication and host survival (Fig. S8D and E), confirming that TubD is dispensable for asexual development. Furthermore, gametocytemia, sex ratio, gametocyte activation, and egress were comparable between ΔtubD and WT parasites (Fig. 7A through D). However, ΔtubD parasites produced 13.7% fewer exflagellated gametes and showed a significantly reduced number of exflagellation centers (6 ± 2 centers in ΔtubD compared to 104 ± 8 centers in WT; Fig. 7E and F). Importantly, *in vitro* ookinete differentiation assays revealed a complete block in mature ookinete formation in ΔtubD cultures (0% formation in ΔtubD versus 75.2% in WT; Fig. 7G and H), with all parasites arrested at the zygote or stage I/II (Fig. 7I).

To elucidate the basis of this developmental arrest, we performed TEM on fixed ookinetes. The architecture of the apical complex and the formation of the IMC appeared to be intact in ΔtubD parasites (Fig. 7J, a, b ,e and f), indicating that TubD is not necessary for the initial biogenesis of the IMC. However, while the SPMTs in WT ookinetes formed ordered and evenly spaced arrays (Fig. 7J, c and d), the ΔtubD parasites displayed severely disorganized microtubule bundles with irregular spacing and alignment (Fig. 7J, g and h).

Together, these data demonstrate that TubD, while dispensable for asexual stage development and IMC formation, is essential for SPMT organization and productive ookinete maturation—phenotypes that resemble those resulting from PbIMC1j deficiency.

## DISCUSSION

This study demonstrated that PbIMC1j is a palmitoylated protein conserved among Apicomplexans. We found that the C-terminal region, which contains the CC domain of PbIMC1j, is required for its function. Specifically, the IMCp domain of PbIMC1j mediates interactions with both ISC1 and TubD. Consistent with the roles of other alveolins such as IMC1b and IMC1h (25, 26), truncation of the C-terminal region of PbIMC1j severely compromises ookinete morphology and motility, correlating with reduced infectivity in both ookinetes and sporozoites. Furthermore, the expression and localization of PbIMC1j are closely associated with those of ISC1 and TubD during the ookinete stage. The phenotypic resemblance between ΔtubD parasites and PbIMC1j-disrupted strains underscores a functional interconnection. Collectively, these findings indicate that PbIMC1j is required for both the asexual stage fitness and mosquito-stage transmission.

The IMC1 family exhibits stage-specific expression, reflecting complex regulatory mechanisms in *Plasmodium* biology (12, 25, 38–40). While IMC1a localizes to sporozoites, and IMC1b and IMC1i are predominant in ookinetes (30), IMC1h is present in both stages, and IMC1g is expressed across asexual stages, gametocytes/gametes, and ookinetes (28, 30). Our work reveals that PbIMC1j is unique in its expression throughout the entire life cycle. Unlike PbIMC1g, which associates with the SPN only in schizonts and mature ookinetes (30), PbIMC1j is SPN-associated during schizogony, in activated gametocytes, and throughout development from zygotes to ookinetes. Its SDS-solubility profile in schizonts and ookinetes, akin to PbIMC1g (30), suggests a role in structural integrity. As palmitoylation governs protein stability, membrane affinity, and function in malaria parasites—as documented for IMC1g, IMC1c, and IMC1a (24, 30), we confirmed PbIMC1j palmitoylation via click chemistry. Treatment with the broad-spectrum inhibitor 2 BP disrupted its subcellular targeting in schizonts. Future mutagenesis studies aimed at identifying specific palmitoylation sites will be essential to elucidate how this modification regulates PbIMC1j targeting and activity.

Deficiency in PbIMC1j leads to pronounced morphological defects, affirming its role as a structural component of the ookinete IMC/SPN, analogous to IMC1b and IMC1h (25, 27). Notably, knockout and C-terminal deletion mutants exhibit a distinctive "bulb-like" morphology and increased susceptibility to osmotic stress, indicating impaired mechanical stability. Ultrastructural analysis revealed disorganized SPMT arrays in both PbIMC1j-KO and -Del ookinetes. Given that SPMTs underpin the IMC in mosquito stages and are essential for structural integrity during invasion (41), their disorganization likely contributes to reduced mechanical resilience. This aligns with reports that null mutants of IMC1a, −1b, and −1 h also exhibit structural vulnerabilities (12, 25, 26). Previous work on PbIMC1h similarly associated mechanical robustness with infectivity (28). Thus, dysregulated SPMTs and consequent mechanical instability likely explain the impaired gliding motility and infectivity of PbIMC1j-KO and -Del mutants.

The IMC1 family constitutes an essential submembrane network that ensures structural integrity (22). The predominant expression of IMC1b, IMC1h, and IMC1j in ookinetes suggests functional synergy as none alone suffices to maintain ookinete morphology (25, 27). This is supported by earlier observations that double deletion of IMC1b and −1 h does not exacerbate morphological defects beyond single knockouts (26). Beyond the overall abundance, the functional integrity of specific domains is crucial. Our study identifies two key elements: the conserved IMCp domain and the C-terminal region. The IMCp domain mediates protein-protein interactions within the SPN, a conserved mechanism among apicomplexans. For example, in *T. gondii*, IMC6 interacts with IMC2, IMC3, and ILP1 via its IMCp domain (22), and heterologous complementation studies demonstrate functional conservation between *T. gondii* IMC6 and *P. berghei* IMC1h IMCp domains (22, 28). In PbIMC1j, this domain mediates critical interactions with partners such as ISC1 and TubD. In contrast, the C-terminal region, predicted to contain a CC domain, is required for proper protein targeting and integration into the IMC/SPN. This is evidenced by the severe phenotypic similarity between full knockout (KO) and C-terminal deletion (Del) mutants, both of which exhibit profoundly disrupted ookinete formation and SPMT organization. The more severe defect in Del mutants compared to the mild impairment in *glmS*-mediated knockdown (~95% reduction) suggests that the truncation may exert a dominant negative effect by producing a misfunctional protein that disrupts the IMC network, whereas the knockdown permits residual function from the full-length protein. The stage-specific discrepancy in Del protein localization—diminished in schizonts yet effectively targeted in ookinetes—implies compensatory mechanisms in the latter stage, possibly through interactions with other ookinete-specific proteins, as observed for CC domains in other apicomplexans (42). Thus, the coordinated action of the N-terminal IMCp interaction domain and the C-terminal targeting region is indispensable for PbIMC1j function.

Our study provides compelling evidence that PbIMC1j interacts with the suture protein ISC1. In *Toxoplasma*, the IMC suture comprises six ISCs and six transverse suture components (TSCs) positioned at alveolar sac junctions (10, 43–45). ISC3 knockout causes severe morphological defects, nuclear segregation defects, and mitochondrial collapse, drastically attenuating virulence (45). A co-immunoprecipitation analysis using ISC4 as bait revealed interactions with IMC10 (45), a *Toxoplasma* homolog of PbIMC1j, reinforcing our findings. We demonstrate that PbIMC1j binds ISC1 via its IMCp domain and that disrupting PbIMC1j reduces ISC1 expression, underscoring their functional relationship. Among ISCs, only ISC1 and ISC3 have homologs in *Plasmodium* (45). Although ISC1 is predicted to be essential in *P. falciparum* (MFS: −2.623; MIS: 0.239) (46), its specific role in *Plasmodium* species remains to be fully elucidated. Understanding these interactions may yield critical insights into parasite biology and inform therapeutic strategies.

We also identified an interaction between PbIMC1j and TubD. Tubulins are ancient GTPases critical for mitosis and, as SPMTs, for defining zoite shape and rigidity (47–50). *Plasmodium* species contain single homologs of δ- and ε-tubulin (19). Its δ-tubulin (TubD) features a unique “AGGSGSG” motif, diverging from the canonical tubulin signature “(S/A/G)-G-G-T-G-(S/A)-G” (51). Although TubD was previously thought to be microgamete-specific, we detected its expression in *P. berghei* ookinetes (51). Pull-down assays confirmed that TubD binds the PbIMC1j IMCp domain. Disruption of PbIMC1j expression or its C-terminus significantly reduced TubD levels, indicating co-dependency. Accordingly, ΔtubD ookinetes arrest early at stages I/II with disorganized microtubules, phenocopying PbIMC1j disruption. This morphological defect is inconsistent with a nucleation deficiency, which would be expected to prevent microtubule formation altogether rather than yield disorganized arrays. Given that γ-tubulin depletion abolishes SPMT formation (52) and studies in *Toxoplasma gondii* have established that SPMTs and the inner membrane skeleton are co-dependent for their biogenesis (52), we propose that PbIMC1j functions as a microtubule-associated protein (MAP) that cooperates with TubD to organize SPMTs in ookinetes.

Several MAPs have been identified in *Toxoplasma*, including SPM1/2, TrxL1, TLAP1-4, AC9, and AC10 (53–56). Most of these proteins have homologs in *Plasmodium* species. However, due to the differences in the life cycles of *Toxoplasma* and *Plasmodium*, MAPs in *Plasmodium* may serve distinct functions. Recently, SPM3 was shown to anchor SPMTs to the IMC in *Plasmodium*, and its deletion reduces the transmission efficiency (57). Additionally, IMC-anchored ISP proteins (ISP1 and ISP3) in *P. berghei* interact with β-tubulin to maintain SPMT integrity and support zygote-to-ookinete differentiation (5). Our study is the first to identify an IMC1 family member, IMC1j, as a TubD-interacting partner that maintains SPMTs and enables ookinete development. Interestingly, the deletion of PbIMC1j results in issues for the ookinetes, but not for the gametocytes, whereas the deletion of TubD affects both stages. These differences may arise from the low expression levels and non-functionality of the IMC1j protein in gametocytes and gametes. Given the functional redundancy of MAPs, other proteins may regulate TubD for SPMT organization during gametocyte development. The precise architecture and interplay between SPMTs and the IMC remain an active area of investigation, and defining TubD’s role will advance our understanding of the cytoskeletal interplay in apicomplexans.

In conclusion, we have identified PbIMC1j as a ubiquitously expressed alveolin that is most abundant in ookinetes. This protein plays multiple roles in enhancing fitness during the asexual stage, influencing ookinete morphology, providing mechanical stability, facilitating gliding motility, and affecting the infectivity of both ookinete and sporozoite stages. Our findings emphasize the importance of the C-terminal domain for proper targeting of SPN/IMC and overall protein function, while the IMCp domain is crucial for mediating interactions with core IMC and SPMT components. These characteristics highlight PbIMC1j as a promising therapeutic target. Furthermore, although the deletion of PbIMC1j significantly hinders sporozoite accumulation in the salivary glands, the exact nature of the defect—whether it occurs during oocyst formation, sporozoite development, or salivary gland invasion—remains unclear. Investigating the mechanistic basis of this phenotype is an important direction for future studies focused on the stage-specific roles of the IMC and its associated proteins.

## MATERIALS AND METHODS

### Sequence analysis

The domain predictions for the PbIMC1j protein (PlasmoDB ID: PBANKA_1120400) were analyzed using SMART (https://smart.embl.de) and HHPred server (https://toolkit.tuebingen.mpg.de/tools/hhpred) (58). Potential palmitoylation sites were identified with CSS-Palm 4.0 software (59). Potential N-glycosylation sites were predicted using the NetNGlyc 1.0 online tool (https://services.healthtech.dtu.dk/services/NetNGlyc-1.0/) (60). The sequences of IMC1j protein orthologs from different organisms, including *P. falciparum* (PF3D7_0621400), *P. vivax* (PVX_114190), *P. chabaudi* (PCHAS_1119900), *P. yoelii* (PY17X_1121700), *T. gondii* (XP_002367942.1), *Eimeria tenella* (XP_013233775.1), and *Cystoisospora suis* (XP_067926278.1), were obtained from PlasmoDB or NCBI databases. Multiple sequence alignments and phylogenetic trees for these IMC1j orthologs were conducted using MEGAX, ESPript 3.0, and Evolview (61–63).

### Parasites, mice, and mosquitoes

Animal experiments were conducted followed approved protocols (CMUXN2023002) from China Medical University’s ethics committee. Female 6–8 weeks Kunming mice (Huafukang Biotechnology Co., Beijing, China) were used for parasite propagation, transfection, phenotype analysis, and direct mosquito feeding assays. To analyze host immune responses, female 6–8-week C57BL/6J and BALB/c mice (Huafukang Biotechnology Co.) were infected with 10^6^ *Pb*. ANKA parasites *via* intraperitoneal (i.p.) injection. Parasitemia was checked with Giemsa-stained smears, and mouse survival was recorded daily. Female *Anopheles stephensi* mosquitoes were provided with a 10% sucrose solution and maintained at 25°C with 75% humidity as described (64).

### Plasmid construction

The pYCm plasmid was used to edit the gene of the *Plasmodium berghei* ANKA strain (*Pb*. ANKA). Two plasmids were generated: pYCm-PbIMC1j-HA-*glmS*, which is for C-terminal tagging of the PbIMC1j protein, and pYCm-PbIMC1j-Del, intended for disrupting the C-terminal portion (amino acid 272–623) of the PbIMC1j protein. To create these plasmids, we amplified the homologous recombination arms containing restriction sites (NcoI and SacII) along with overlapping sequences necessary for recombinant integration into the pYCm plasmid using specific primers (Table S3). The fragments were then purified and ligated into the pYCm vector between the HindIII and AflII restriction sites. Next, the 3× HA *glmS* and 2× Myc fragments were ligated between the NcoI and SacII sites in-frame with the *pbimc1j* gene. The specific guide RNAs (sgRNAs) used in this study were designed using the EuPaGDT online tool (65), synthesized in a thermal cycler (Bio-Rad, CA, USA) with a gradient cooling program, and subsequently ligated into the BsmBI site of the pYCm vector.

To knockout the *pbimc1j* gene, we amplified two DNA fragments: a 524 bp 5’ recombinant fragment (5R, spanning from −506 to −18 bp) and a 470 bp 3’ recombinant fragment (3R ranging from +1 to +470 bp) of the *pbimc1j* gene using specific primers (Table S3). The PCR fragments were then purified and inserted into the pL0034 vector at designated restriction sites (HindIII/ApaI for 5R and XhoI/NotI for 3R), creating the pL0034-PbIMC1jKO. Similarly, to knockout the *tubD* gene (PlasmoDB ID: PBANKA_0834600), we amplified the 5R fragment (−605 to 3 bp) and the 3R fragment (+1 to +642 bp) of the *tubD* gene from *P. berghei* gDNA. After purification, these fragments were ligated into the HindIII/ApaI and XhoI/NotI sites of the pL0034 plasmid, respectively, generating the pL0034-TubDKO plasmid.

For prokaryotic system expression using *Escherichia coli* (*E. coli*), DNA segments coding for residues Y_248_-Y_387_ of inner membrane suture protein 1 (ISC1, PBANKA_1354600) and residues Y_257_-K_454_ of the δ-tubulin (TubD) were amplified using *P. berghei* cDNA as the template (Table S3) and were subsequently inserted into the pET32a plasmid.

To express the IMCp domain (K_134_-K_228_) of PbIMC1j, the coding sequences were synthesized and cloned into the pCDNA3.1-N-FLAG vector (BGI Genomics Co., Beijing, China) using HindIII and NotI sites. The coding regions for ISC1 and TubD were optimized for mammalian cell expression and inserted into the pCDNA3.1(+)-Myc-HisA plasmid (BGI Genomics Co.) using NotI and XhoI sites.

All PCRs and plasmid constructions were conducted using enzymes purchased from Vazyme (Nanjing, China).

### Recombinant protein expression and antiserum generation

For recombinant protein expression, the vectors pET32a-ISC1 and pET32a-TubD were first transformed into *E. coli* Rosetta (DE3) super competent cells (Beyotime, Shanghai, China) and then induced with 1 mM IPTG at 21°C for 24 hours. The His-tagged ISC1 and TubD proteins were purified using BeyoGold His-tag Purification Resin (Beyotime). After purification, the purity and specificity of the recombinant proteins were assessed using Coomassie Blue Staining and immunoblotting, respectively. To generate antigen-specific antiserum, 50 µg of recombinant ISC1 or TubD proteins was mixed with complete Freund’s adjuvant (Sigma-Aldrich, VA, USA) and inoculated into BALB/c mice. Three booster injections were administered at 2-week intervals, using 25 µg of protein mixed with incomplete Freund’s adjuvant (Sigma-Aldrich). The antigen-specific sera were collected 10 days post the final immunization.

### Cell culture, transfection, cloning, and diagnostic PCR

HEK293T cells were grown in DMEM supplemented with 10% fetal bovine serum (FBS) at 37°C and under 5% CO_2_ conditions. To express fusion proteins, the pCDNA3.1-N-Flag and pCDNA3.1-Myc-His vectors were co-transfected into the cells using jetPRIME (Polyplus, Strasbourg, France). After transfection, the cells were harvested, lysed, and analyzed 24 or 36 hours later.

Transfection of the *Pb*. ANKA strain was conducted as described (66). In summary, cardiac blood was collected from parasite-infected mice exhibiting 3%–5% parasitemia. This blood was cultured in a schizont culturing medium (RPMI 1640, 50 mg/L penicillin, 50 mg/L streptomycin, and 20% FCS) for 12 to 15 hours at 37°C. Matured schizonts were isolated using a 55% Nycodenz gradient and mixed with 100 µL of incomplete cytomix (120 mM KCl, 0.15 mM CaCl_2_, 2 mM EGTA, 5 mM MgCl_2_, 10 mM K_2_HPO_4_/KH_2_PO_4_, and 25 mM HEPES) containing 15 µg of the relevant plasmid. Electroporation was carried out using the Nucleofector 2B (Lonza, Basel, Switzerland), and the mixture was immediately injected intravenously into Kunming mice (67). One day later, the mice were treated with pyrimethamine (Sigma-Aldrich), and blood samples were collected for genomic DNA extraction using the BeyoMag Blood Genomic DNA Isolation Kit (Beyotime). The successful genomic editing of the target locus was checked with diagnostic PCR using specific primers (Table S3). Limiting dilution was performed to isolate single clones with the desired modification for further phenotypic analysis.

### *P. berghei* culture and stage-specific purification

Gametocytes and ookinetes were purified as described (66). To purify gametocytes, mice were first treated with 70 µg/g of phenylhydrazine (PHZ, Sangon Biotech, Shanghai, China) 3 days before being infected with 10^8^ parasites. At day 3 post-infection (p.i.), the infected mice were given drinking water containing 20 mg/L sulfadiazine (Sigma-Aldrich) for 48 hours to eliminate asexual stage parasites. Afterward, gametocytes were purified using a 48% Nycodenz gradient. Once purified, the gametocytes were placed in an ookinete culture medium (OCM, RPMI 1640, 20% FCS, and 6 U/mL heparin, pH 8.0) at 25°C for 15 minutes for activation. To isolate ookinetes, parasites were collected at day 3 p.i., cultured in an OCM at 19°C for 24 hours, and then isolated using a 62% Nycodenz gradient.

### Immunofluorescence assay

Parasites were fixed at room temperature (RT) for 30 minutes using a fixation buffer (4% formaldehyde and 0.0075% glutaraldehyde), as described (68). After fixation, the samples were permeabilized on ice with 0.1% Triton X-100 for 10 minutes and then neutralized with sodium borohydride (0.1 mg/mL). Following neutralization, the samples were blocked using SuperBlock (ThermoFisher) for 10 minutes at RT and then incubated with primary antibodies, which included mouse anti-HA mAb (Abcam, Cambridge, UK) and mouse anti-Myc mAb (ThermoFisher). Additionally, various rabbit antibodies produced in our lab (30) were used, including anti-MSP1 serum, anti-α-tubulin II serum, anti-G377 serum, anti-P25 serum, and anti-PP6 serum. Subsequently, the primary antibodies were detected using species-specific secondary antibodies conjugated with fluorescent dyes Alexa Fluor 488 and 555 (ThermoFisher). The stained samples were placed on coverslips, mounted with a DAPI-containing antifade solution (ThermoFisher), sealed, and visualized using a Leica STELLARIS 5 confocal microscope (Leica, Wetzlar, Germany) with consistent imaging settings.

### Immunoblot analysis

For Western blotting, lysates from 1 × 10^7^ parasites were loaded onto SDS-PAGE for protein separation and then transferred to PVDF membranes (Millipore, MA, USA). A SuperBlock solution was used to prevent nonspecific binding (ThermoFisher). Then, the membranes were probed with primary antibodies, including mouse anti-HA mAb (PTM), rabbit anti-Myc (PTM), anti-his tag mAb (PTM), anti-Flag mAb (PTM), or anti-ISC1 sera, anti-TubD sera, anti-MyoA sera (Genscript, Nanjing, China), mouse anti-β-actin (PTM), mouse anti-GAPM2 sera (30), and mouse anti-GAPDH (PTM) for 1 h at RT. Following three rinses with TBS-T, the membranes were probed with corresponding HRP-conjugated secondary antibodies (ThermoFisher). The membranes were visualized using the SuperSignal West Atto Ultimate Sensitivity Substrate (ThermoFisher) and imaged on a Tanon 4200 (Tanon, Shanghai, China). The signal intensity of the target protein bands was measured with ImageJ software (69).

### Protein solubility assay

To analyze the protein solubility, we followed a specific procedure (70). In brief, purified schizont and ookinete stage PbIMC1j^HA-^*^glmS^* parasite (1 × 10^6^ cells) underwent three freeze-thaw cycles in PBS containing 1% protease inhibitor (ThermoFisher). The supernatant containing the cytosolic soluble proteins, referred to as FZ, was collected after centrifugation at 4°C for 5 minutes at 13,000 *g*. The pellet was thoroughly rinsed thrice with PBS and then subjected to additional extraction using 1% Triton X-100 for 30 minutes on ice to isolate the transmembrane proteins. The insoluble fraction from the Triton X-100 extraction was further extracted using 2% SDS for 30 minutes at RT to obtain membrane-associated proteins.

### Click chemistry analysis

For the click chemistry assay (30), schizonts or ookinetes were resuspended in a culture medium containing 100 µM of alkynyl palmitic acid (Alk-C16, TargetMol, MA, USA) and then incubated at 37°C for 4 hours. Following the incubation, the labeled parasites were rinsed with PBS and prepared for immunoblot analysis. A click chemistry reaction was performed using a 30 µL cocktail containing biotin azide (ThermoFisher), copper (II) sulfate, and a reducing agent at RT for 90 minutes. The resulting mixture was treated with methanol, chloroform, and water, followed by centrifugation. After discarding the upper aqueous layer, the remaining samples were treated with cold methanol and then pelleted by centrifugation. The labeled samples were air-dried and configured in an immunoprecipitation solution (50 mM Tris, 150 mM NaCl, and 1% SDS) for precipitation with streptavidin beads (ThermoFisher). After washing, the captured proteins were eluted with 2 × SDS buffer and analyzed by immunoblotting.

An IFA analysis was conducted to study the localization of palmitoylated proteins in schizonts and ookinetes. The schizonts or ookinetes were labeled with Alk-C16, followed by fixation and permeabilization. A click chemistry reaction (biotin-azide, 1 × Click-iT reaction buffer, CuSO_4_ solution, and a buffer additive) was performed for 30 minutes at RT. The subsequent IFA analysis was performed as described and detected using a Leica STELLARIS 5 confocal microscope (Leica). The antibodies used in the analysis included streptavidin-FITC (ThermoFisher), mouse anti-HA mAb (Abcam), and rabbit anti-MSP1 or rabbit anti-P25. Additionally, Alexa Fluor 647 chicken anti-rabbit IgG antibodies (ThermoFisher) and Alexa Fluor 555 goat anti-mouse IgG antibodies (ThermoFisher) were used for detection.

### Analysis of parasite phenotypes

In evaluating gametocytogenesis, a dose of 10^8^ parasites was administered intraperitoneally to PHZ (Sangon Biotech) pretreated mice. Subsequent analysis of gametocytemia and the male-to-female ratios was carried out at day 3 p.i. For the exflagellation assay, a mixture was prepared by combining mouse tail blood with OCM at a 1:4 ratio for 15 minutes at 25°C. Using a light microscope, the number of exflagellation centers was counted at 400 × magnification.

The conversion rate of mature ookinetes was determined according to established protocols (66). Briefly, 10 µL of parasite-infected mouse blood with a gametocytemia of 8%–10% was mixed with 90 µL of OCM at 20°C for 24 hours. Following incubation, the ookinetes were stained with an anti-Pbs21 mAb. The matured ookinete conversion rate was calculated by dividing the number of Pbs21-positive crescent-shaped ookinete (Stage V) by the total number of Pbs21-positive ookinetes (encompasses stages I through V). To evaluate the influence of the protein palmitoylation inhibitor 2‐BP (Sigma-Aldrich) on the ookinete conversion process, 2‐BP (100 µM) was introduced to the in *vitro* ookinete cultures 1 hour post-fertilization. For genetic knockdown via the *glmS*-riboswitch system, the transgenic parasite line PbIMC1j^HA-^*^glmS^* was exposed to 2.5 mM glucosamine (GlcN) at the initiation of *in vitro* culture and maintained under treatment for 24 hours.

In the mosquito transmission analysis, a cohort of 30 to 50 *An. stephensi* mosquitoes were permitted to feed on anesthetized parasite-infected mice for 1 hour, ensuring a comparable level of gametocyte presence, as verified by Giemsa-stained blood smears. Subsequently, the mosquitoes were dissected on day 10 post the direct feeding assay (DFA) and stained with 0.5% mercurochrome. Oocyst quantification was performed using a Nikon microscope with a 63× oil immersion objective.

For the enumeration of salivary gland sporozoites, mosquitoes were dissected on day 19 post-DFA, allowing for the computation of the average sporozoite count per mosquito. In the sporozoite invasion assay, 3,000 sporozoites were administered to BALB/c mice, and the parasite’s transmission capability was monitored daily through Giemsa-stained blood smears for 20 days.

### Flow cytometry analysis

Flow cytometry was employed to analyze splenocyte populations. Initially, 2 × 10^6^ splenocytes were stimulated for 5 hours with PMA (200 ng/mL) and ionomycin (1 µg/mL), with Brefeldin A (BD Biosciences, NJ, USA) added to block cytokine release. After stimulation, the cells were stained for surface proteins and then fixed and permeabilized using the BD Cytofix/Cytoperm kit (BD Biosciences). Subsequently, intracellular staining was performed to evaluate the expression of various molecules. The T cells gathering in the brain were analyzed as described (71). Briefly, the brains were excised and gently homogenized in RPMI 1640 containing 2% fetal calf serum (FCS). The resulting homogenate was layered over a 30% Percoll gradient (Dingguo, Beijing, China) and centrifuged for 25 minutes at 2,500 × *g*. Lymphocytes were collected and analyzed via cell surface immunofluorescence staining. The flow cytometric analysis used several antibodies, including BV605-CD4 (Clone GK1.5, Biolegend), BV510-CD8 (Clone 53-6.7, Biolegend, CA, USA), APC-IFN-γ (Clone XMG1.2, BD), Percp-cy5.5-T-bet (Clone 4B10, Biolegend), PE-granzyme B (Clone QA16A02, Biolegend), APC-Cy7-CD45 (Clone 30-F11, BD), and FITC-CD11b (Clone M1/70, Biolegend). Data were acquired using a BD FACS Celesta cytometer (BD Biosciences) and analyzed using FlowJo v10 software (Tree Star, OR, USA).

### Ookinete gliding assay

Extended ookinete cultures were mixed with Matrigel (BD Biosciences) while on ice. This mixture was then placed onto a slide, followed by covering, sealing with nail varnish, and resting for 30 min prior to observation at 19°C. After locating a gliding ookinete, time-lapse videos were captured, taking one frame every 20 seconds for 30 min using a Leica STELLARIS 5 confocal microscope. The motility speeds of the observed ookinetes were calculated using ImageJ software, employing the Manual Tracking plugin for precision in data analysis.

### Osmotic shock and viability assays

Hypo-osmotic shock assays were performed by mixing the ookinetes with an equal volume of water, decreasing the osmotic strength by half. After 5 minutes of incubation, standard conditions were restored by adding a 10 × PBS solution. To assess cell viability, we used fluorescence microscopy with 0.5% propidium iodide (ThermoFisher) and 1% Hoechst 33342 (ThermoFisher). Ookinetes were considered nonviable if stained with both dyes and viable if stained only with Hoechst. The viability results were adjusted to reflect 100% based on untreated cells.

### Transmission electron microscopy

The ultrastructure of mutant strains was examined using transmission electron microscopy (TEM) (72). In summary, purified schizonts and ookinetes were fixed in 2.5% glutaraldehyde. After fixation, the samples were treated with osmium tetroxide (OsO_4_), dehydrated in ethanol, and infiltrated with propylene oxide. The samples were subsequently embedded in Spurr’s epoxy resin, sliced into thin sections, and stained with uranyl acetate and lead citrate before examination with a Hitachi transmission electron microscope (Hitachi, Tokyo, Japan) operating at 120 kV.

### Immunoprecipitation and mass spectrometry

A total of 1 × 10^8^ PbIMC1j^HA-^*^glmS^* and parental *Pb*. ANKA ookinetes lysates was extracted using immunoprecipitation (IP) lysis buffer (25 mM Tris [pH 7.5], 150 mM NaCl, 0.1% SDS, 1% NP40) on ice for 30 minutes. The supernatants of the extraction were then subjected to immunoprecipitation using Pierce Anti-HA magnetic beads (ThermoFisher). The beads were washed four times with TBS-T (TBS containing 0.1% Tween-20) and then once with ultrapure water. The proteins bound to beads were eluted at 95°C for 10 minutes using 2 × SDS loading buffer.

For LC-MS/MS analysis, the gel pieces from IP samples underwent short SDS-PAGE migration and were subsequently digested using trypsin proteases. The digested samples were then analyzed using a Thermo Scientific Q Exactive Plus mass spectrometer (ThermoFisher). The resulting data set was processed with Proteome Discoverer 2.4 (ThermoFisher) and searched against the PlasmoDB-59_PbergheiANKA database (Jingjie PTM Biolab Co., Hangzhou, China). The dataset was submitted to the ProteomeXchange Consortium *via* the iProX partner repository under accession number PXD059715 (73, 74). To verify the presence of δ-tubulin and ISC1 in the PbIMC1j-HA immunoprecipitates, additional PbIMC1j-HA and *P. berghei* ookinete lysates were prepared and analyzed using immunoblotting.

For the co-immunoprecipitation (co-IP) analysis, the supernatants from the extracted cell lysates were incubated overnight at 4°C with Flag-, Myc-, or HA Nanobody Magarose Beads (AlphLifeBio, Shenzhen, China). The beads were washed three times with PBS containing 0.1% Tween-20 (PBS-T) and subsequently dissolved in 100 µL of 2 × SDS loading buffer. An equal amount of the supernatant from each sample was used for immunoblotting. The membrane was probed with anti-FLAG mAb (PTM), anti-Myc mAb (PTM), anti-tubulin mAb (PTM), anti-ISC1 serum, or anti-TubD serum and developed as previously described.

### Statistical analysis

To assess the differences between the parental *Pb*. ANKA strain and the mutant strains, as well as among the mutant parasites treated with (+) and (−) glucosamine (GlcN) treatment, we calculated statistical significance using either a two-tailed Student’s *t*-test or a Mann–Whitney *U* test plugin in the GraphPad Prism version 9.0 software (La Jolla, CA, USA).

## SUPPLEMENTAL MATERIAL

Supplemental material mbio.02669-25-s0001.pdf Figures S1-S8, Table S1, and supplemental table and movie captions.

Table S2 mbio.02669-25-s0002.xlsx The interactome of PbIMC1j was characterized using IP/MS.

Table S3 mbio.02669-25-s0003.xlsx IMC1j primer list.

Movie S1 mbio.02669-25-s0004.mp4 The gliding motility of WT *P. berghei* ookinetes.

Movie S2 mbio.02669-25-s0005.mp4 The gliding motility of PbIMC1j KO ookinetes.

Movie S3 mbio.02669-25-s0006.mp4 The gliding motility of PbIMC1j Del ookinetes.

ASM does not own the copyrights to Supplemental Material that may be linked to, or accessed through, an article. The authors have granted ASM a non-exclusive, world-wide license to publish the Supplemental Material files. Please contact the corresponding author directly for reuse.
