# Supplementary material for: Plasmodium berghei IMC1j interacts with δ-tubulin to orchestrate subpellicular microtubule organization in ookinetes
Source: mBio. 2025 Oct 21;16(11):e02669-25. doi: 10.1128/mbio.02669-25 (PMC12607714; doi:10.1128/mbio.02669-25)
Supplement: Supplemental material — Figures S1-S8, Table S1, and supplemental table and movie captions. [file mbio.02669-25-s0001.pdf]

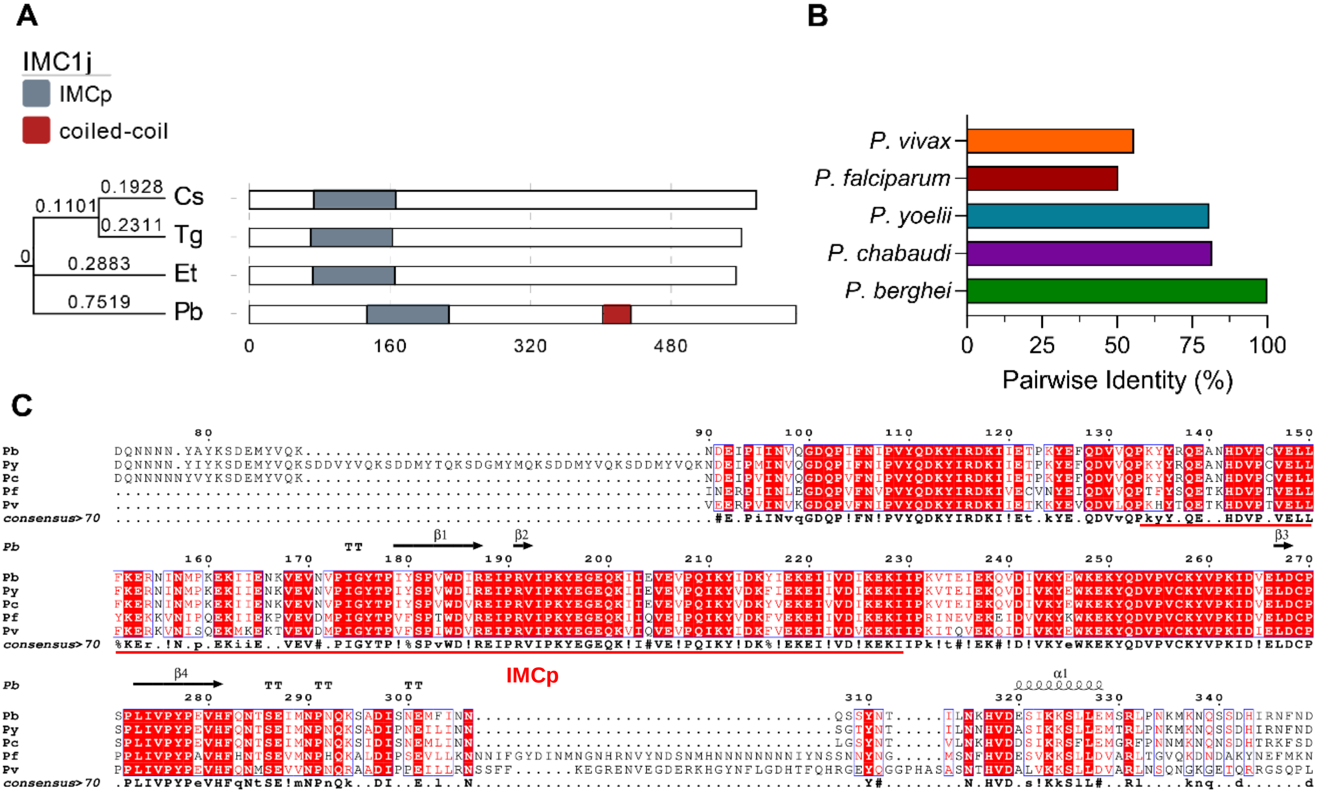

FIG. S1 Sequence alignment of IMC1j protein. (**A**) Phylogenetic tree of PbIMC1j and proteins identified through a BLASTP search across various species. The following IMC1j protein sequences were used: *Cystoisospora suis* (Cs), GenBank no. XP_067926278.1; *Toxoplasma gondii* ME49 (Tg), XP_002367942.1; *Eimeria tenella* (Et), XP_013233775.1; *Plasmodium berghei* ANKA (Pb), PlasmoDB ID: PBANKA_1120400. (**B**) Multiple protein sequences alignment of IMC1j orthologues in *Plasmodium* species. The sequences for *Plasmodium vivax* Sal-I (Pv, PVX_114190), *Plasmodium falciparum* 3D7 (Pf, PF3D7_0621400), *Plasmodium yoelii* 17X (Py, PY17X_1121700), *Plasmodium chabaudi chabaudi* (Pch, PCHAS_1119900), and *Plasmodium berghei* ANKA (Pb) were obtained from PlasmoDB database. (**C**) Sequence alignment of the IMCp domain within IMC1j protein sequences from Pb, Py, Pc, Pf, and Pv were performed using MUSCLE. The predicted IMCp domain of the IMC1j protein is indicated with a red line.

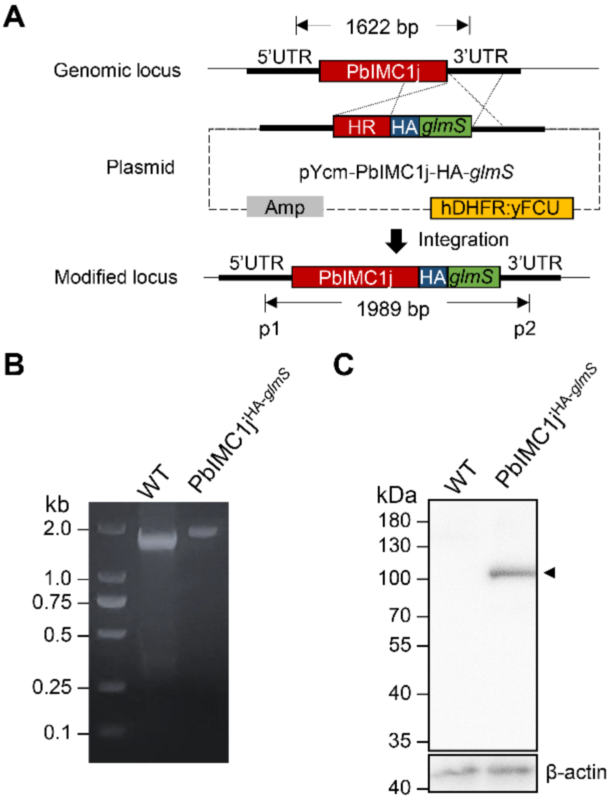

FIG. S2 Generation of the PbIMC1j$^{\text{HA-}\textit{glmS}}$ transgenic parasite line. (**A**) The diagram illustrates the strategy for fusing the C-terminal region of PbIMC1j with a triple hemagglutinin tag (3×HA) and a ribozyme sequence (*glmS*). The positions of the diagnostic PCR primers are marked with black arrows. (**B**) PCR results confirm the presence of the wild-type locus (WT, p1+p2) at 1622 bp and the modified PbIMC1j$^{\text{HA-}\textit{glmS}}$ locus (p1+p2) at 1989 bp. (**C**) A western blot analysis was conducted to assess the expression of the PbIMC1j-HA fusion protein in both WT and PbIMC1j$^{\text{HA-}\textit{glmS}}$ lines using an anti-HA mAb. The arrow highlights the PbIMC1j-HA protein. Protein loading for each lane was verified using the anti-β-actin mAb.

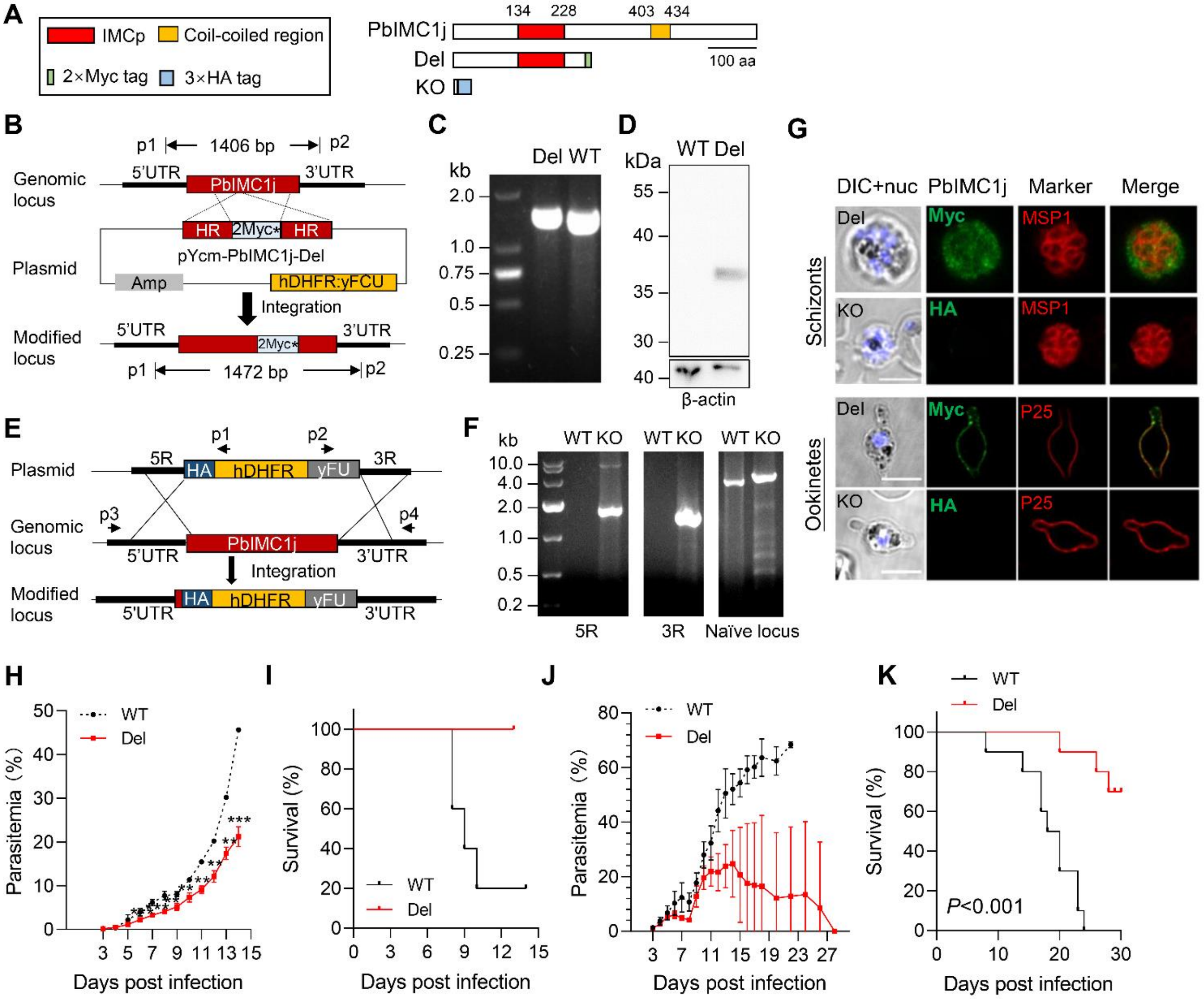

FIG. S3 Generation of PbIMC1j knockout and C-terminal region deletion transgenic parasites. (**A**) Schematic diagrams of PbIMC1j^{KO} (KO) and PbIMC1j^{Del} (Del) proteins. (**B**) A schematic representation of the deletion of the C-terminal region of the PbIMC1j protein. The remaining protein is fused with a Myc-tag using a double crossover strategy. The arrows indicate the primers used for diagnostic PCR. (**C**) Confirmatory PCR results for the PbIMC1j^{Del} (Del) line. The wild-type (WT) locus (primers p1+p2), 1406 bp; the modified locus for Del (p1+p2), 1472 bp. (**D**) The expression of the PbIMC1j-Del protein was evaluated via western blot analysis using an anti-Myc mAb. The arrow indicates PbIMC1j-Del protein (MW: 35.0 kDa). β-actin was employed as a loading control. (**E**) A schematic figure of the double crossover technology used to delete the *pbimc1j* gene is presented. Arrows indicate the primers used for diagnostic PCR. (**F**) Diagnostic PCR confirmed successful integration using genomic DNA from a clonal PbIMC1j knockout (KO) line C1 and WT parasites—the naive locus (p3+p4): WT, 3759 bp; KO, 4790 bp. 5R (p3+p1): WT, null; KO, 1874 bp. 3R (p2+p4): WT, null; KO, 1532 bp. The red arrows indicate the target bands. (**G**) IFA analysis of Del and KO parasites at schizont (upper panel) and ookinete (lower panel) stages detected using anti-Myc and anti-HA mAb, respectively, and co-stained with anti-MSP1 and anti-P25 serum, respectively. Scale bar: 5 µm. (**H-K**) The parasitemia (**H, J**) and survival rate (**I, K**) of WT and Del parasites were monitored daily for 14 and 30 days in C57BL/6 and BALB/c mice. For panels (**H**) and (**J**), parasitemias are expressed as the mean ± SEM. Each group consisted of ten mice, and representative results from three biological replicates are shown. Statistical comparisons between the WT and Del groups were conducted using Kaplan–Meier survival analysis.

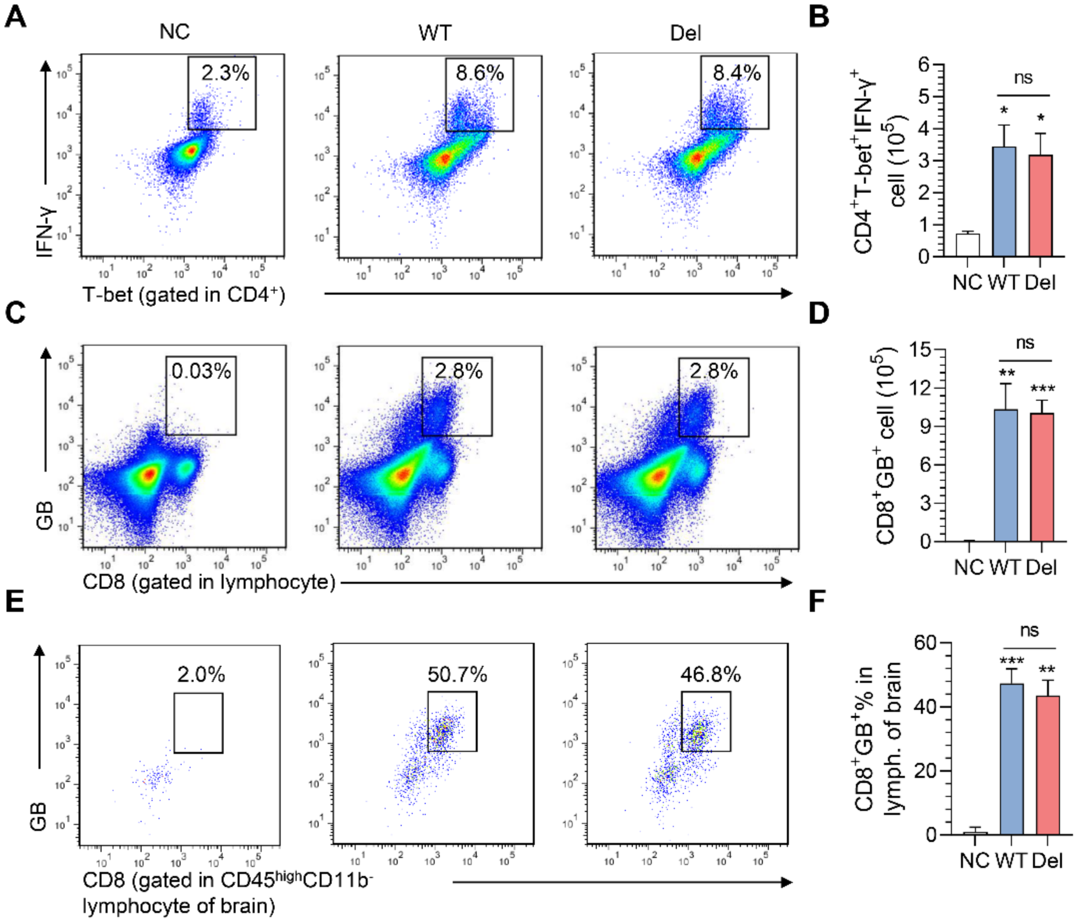

FIG. S4 T cell differentiation following infection with the parental *P. berghei* ANKA and Del strains. Representative plots display the frequencies of splenic (**A**) Th1 cells ($CD4^+T\text{-}bet^+IFN\text{-}\gamma^+$), (**C**) $CD8^+$ T cells ($CD8^+GB^+$), and (**E**) brain $CD8^+$ T cells ($CD8^+GB^+CD45^{high}CD11b^-$). Bar graphs illustrate the quantities of splenic (**B**) Th1 cells ($CD4^+T\text{-}bet^+IFN\gamma^+$) and (**D**) $CD8^+$ T cells ($CD8^+GB^+$), as well as the proportion of brain $CD8^+$ T cells ($CD8^+GB^+CD45^{high}CD11b^-$) shown in (**F**). Lymph, lymphocyte. Data are presented as mean ± SD (n = 3 per group). Significant differences for Del/WT compared to na ïve mice (NC) are indicated (*, $P < 0.05$; **, $P < 0.01$; ***, $P<0.001$; Student's *t*-test), while Del vs. WT was not significant (ns). Data are representative of three independent experiments.

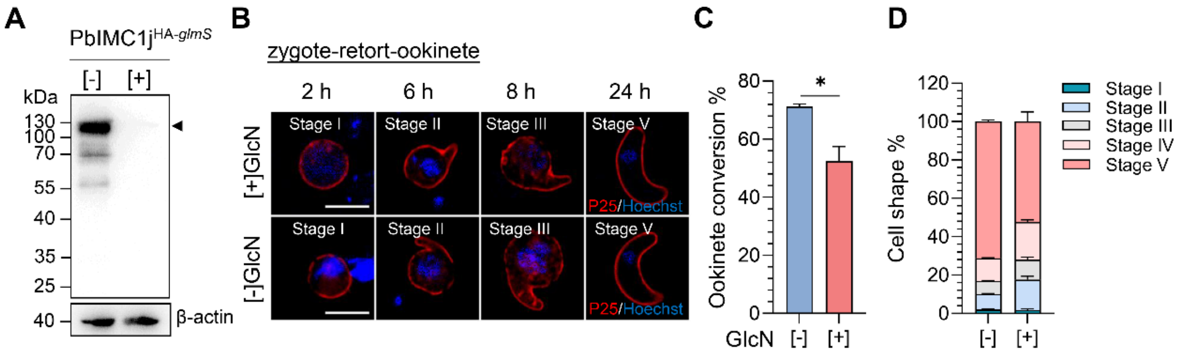

FIG. S5 PbIMC1j is essential for ookinete conversion and motility. (**A**) Western blot analysis indicates reduced levels of PbIMC1j-HA protein when treated with [+] or without [-]GlcN. Samples were collected 24 hours post in *vitro* ookinete culture for detection with anti-HA mAb. β-actin was used as a loading control. (**B**) The developmental time course of PbIMC1j$^{HA\text{-}glmS}$ parasites with [+] and [-] GlcN from 2 to 24 hours post fertilization is depicted, with ookinetes stained using anti-P25 serum (red) and Hoechst 33342 (blue). Scale bar = 5 µm. (**C**) The ookinete conversion rate in PbIMC1j$^{HA\text{-}glmS}$ parasites with [+] and [-] GlcN is presented. The conversion rate of mature ookinetes was calculated based on the percentage of Pbs21-positive parasites that formed typically 'banana-shaped' ookinetes 24 hours after initiating ookinete culture. *, $P < 0.05$ (Student's *t*-test). (**D**) The percentage of parasites exhibiting various morphologies is presented. Data are representative of three independent experiments.

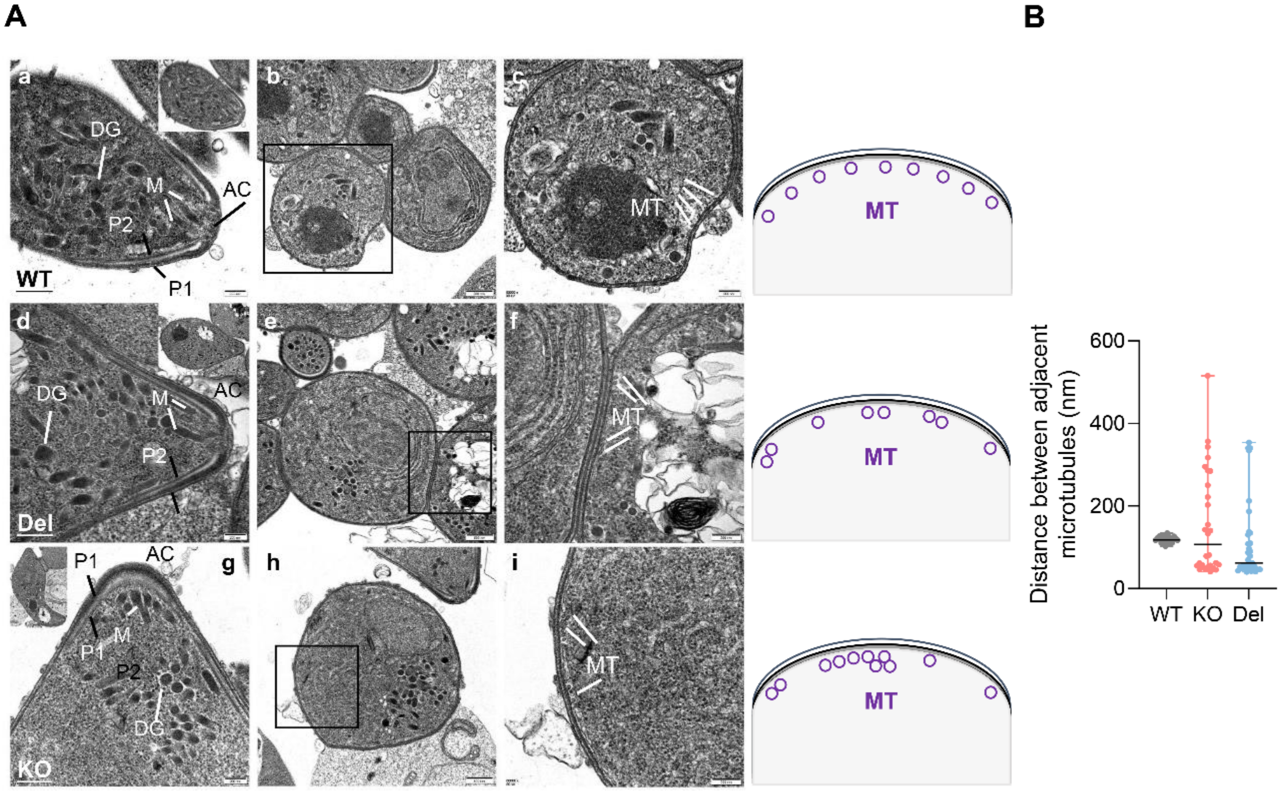

FIG. S6 Ultrastructure analysis of PbIMC1j KO and Del ookinetes. (**A**) TEM images of WT, KO, and Del ookinetes are shown. Longitudinal sections depict a WT ookinete (**a**), a Del ookinete (**d**), and a KO ookinete (**g**). Key structures are labeled as follows: AC, apical complex; M, microneme; DG, dense granule; P1 and P2 represent polar rings 1 and 2, respectively. Cross-sectional views through the periphery of the anterior complex are displayed for WT (**b-c**), Del (**e-f**), and KO (**h-i**) parasites, highlighting subpellicular microtubules (MT). Scale bars are 500 nm for images (**b**, **e**, **h**) and 200 nm for images (**a**, **c**, **d**, **f**, **g**, **i**). Diagrams illustrating the apical complex and the distribution of microtubules in WT, KO, and Del parasites are presented in the right panel. (**B**) The distances of microtubules in WT, KO, and Del ookinetes are presented as Median with ranges derived from two independent experiments (ookinetes number: WT, n = 34; KO, n = 28; Del, n = 33). Data are representative of two biological replicates.

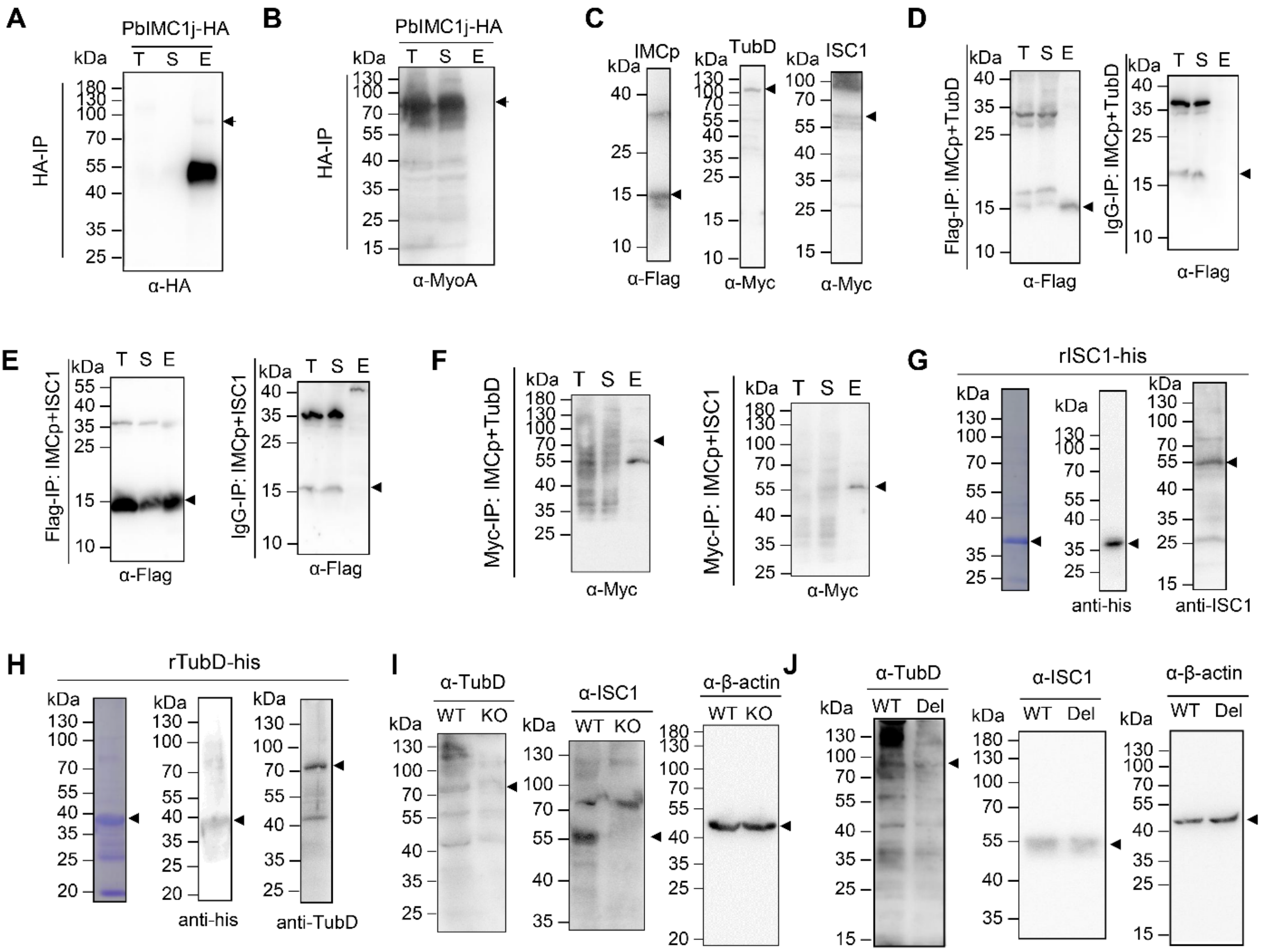

FIG. S7 Interactions between PbIMC1j, ISC1, and TubD proteins. (**A**) Co-immunoprecipitation (co-IP) of PbIMC1j-HA from parasite lysates using HA-conjugated beads, followed by immunoblotting with anti-HA. Arrow indicates PbIMC1j-HA. (**B**) Immunoblot of PbIMC1j-HA precipitates probed with anti-MyoA antisera. Arrow indicates Myosin A protein (92.3 kDa). (**C**) Expression of recombinant Flag-IMCp (13.8 kDa), TubD-Myc-His (85.7 kDa), and ISC1-Myc-His (60.2 kDa) in transfected cells, detected with anti-Flag and anti-Myc. (**D**, **E**) Pull-down assays using anti-Flag or control IgG beads with lysates co-expressing Flag-IMCp and TubD-Myc-His (**D**) or ISC1-Myc-His (**E**). Blots were probed with anti-Flag. T, total input; S, supernatant; E, elution. (**F**) Reciprocal pull-down using anti-Myc beads with lysates containing Flag-IMCp and TubD-Myc-His (left) or ISC1-Myc-His (right). Blots were probed with anti-Myc. (**G**, **H**) Purified recombinant ISC1-His (**G**; 35.4 kDa) and TubD-His (**H**; 41.5 kDa) analyzed by Coomassie staining (left), anti-His immunoblot (middle), and immunoblot of mixed-blood-stage parasite extracts with specific antisera (right; native ISC1: 56.5 kDa; TubD: 82.1 kDa). (**I**, **J**) Western blot analysis of TubD, ISC1, and β-actin (loading control) in PbIMC1j-KO (**I**) and -Del (**J**) mutant parasites. Arrowheads indicate endogenous protein bands. All data are representative of three independent experiments.

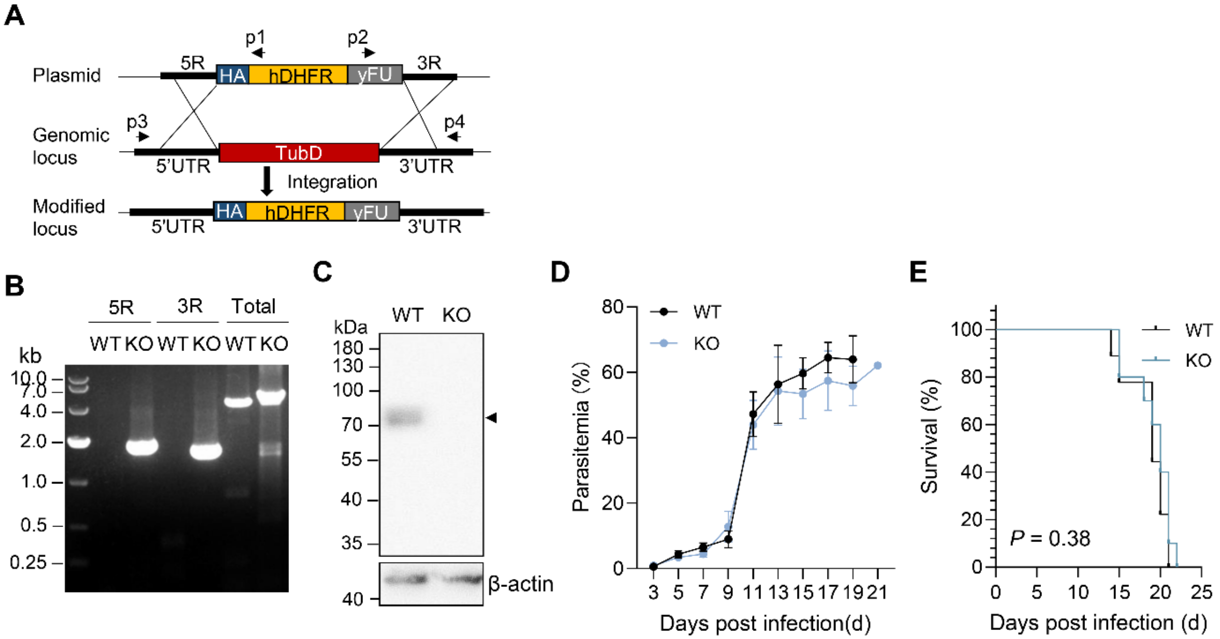

FIG. S8 Generation of ΔtubD transgenic parasites. (**A**) A schematic representation of the ΔtubD strain. (**B**) Confirmatory PCR results for the wild-type (WT) *P. berghei* ANKA and ΔtubD strain (KO). The naive locus (p3+p4): WT, 3821 bp; KO, 4523 bp. 5R (p3+p1): WT, null; KO, 1641 bp. 3R (p2+p4): WT, null; KO, 1498 bp. (**C**) The successful deletion of the *tubD* gene in the ΔtubD strain was also tested by western blot analysis using an anti-TubD serum. The arrow indicates the TubD protein (MW: 82.0 kDa). β-actin was employed as a loading control. Date are representative of three biological replicates. (**D-E**) The parasitemia (**D**) and survival rate (**E**) of WT and ΔtubD parasites were monitored daily for 30 days in BALB/c mice. The parasitemia is presented as the mean ± SD. Each group consisted of ten mice, and representative results from three biological replicates are shown. Statistical comparisons between the WT and ΔtubD groups were conducted using Kaplan–Meier survival analysis.

Table S1. CSS-Palm software predicted palmitoylation sites in PbIMC1j.

Position	Peptide	Score	Cutoff
6	*****MENKQCKLIFSDCCKG	0.8361	0.7766
13	NKQCKLIFSDCCKGRENVAYN	0.8408	0.7766
14	KQCKLIFSDCCKGRENVAYNV	0.8275	0.7766
146	YRQEANHDVPCVELLFKERNI	0.7769	0.7766
257	WKEKYQDVPVCKYVPKIDVEL	0.8141	0.7766
370	NKKNKKMWPFCTFNNCINNET	0.8090	0.7766

Note: The putative palmitoylated sites are labeled with red letters.

Table S2. The interactome of PbIMC1j was characterized using immunoprecipitations followed by mass spectrometry analysis (IP/MS). (A) Proteins identified as interactors of PbIMC1j through immunoprecipitation are listed. WT, wild-type *P. berghei*; IMC1j, PbIMC1j$^{HA\text{-}glmS}$ parasites. **(B)** PbIMC1j unique interactions. These include proteins with at least one unique peptide from the PbIMC1j-HA immunoprecipitation and none from the control (parental parasites). Proteins known to localize to the IMC/SPN or cytoskeletal are highlighted in red.

Table S3. Oligonucleotides in this study.

Movie S1. The gliding motility of wild-type (WT) *P. berghei* ookinetes.

Movie S2. The gliding motility of PbIMC1j knockout (KO) ookinetes.

Movie S3. The gliding motility of PbIMC1j C-terminal region deletion (Del) ookinetes.
